# The role of a mindful movement-based program (Movimento Biologico) in health promotion: results of a pre-post intervention study

**DOI:** 10.3389/fpubh.2024.1372660

**Published:** 2024-06-11

**Authors:** Stefano Spaccapanico Proietti, Manuela Chiavarini, Francesco Iorio, Livia Buratta, Giancarlo Pocetta, Roberta Carestia, Camilla Gobbetti, Chiara Lupi, Antonio Cosenza, Guglielmo Sorci, Claudia Mazzeschi, Andrea Biscarini, Chiara de Waure

**Affiliations:** ^1^Department of Medicine and Surgery, University of Perugia, Perugia, Italy; ^2^Department of Biomedical Sciences and Public Health, Section of Hygiene, Preventive Medicine and Public Health, Polytechnic University of the Marche Region, Ancona, Italy; ^3^Department of Philosophy, Social Sciences and Education, University of Perugia, Perugia, Italy

**Keywords:** mindful movement, psychological well-being, mental health, sense of coherence, interoceptive awareness, young adults, Movimento Biologico

## Abstract

**Introduction:**

Mindful movement is a comprehensive approach that integrates various bodily, emotional and cognitive aspects into physical activity, promoting overall well-being. This study assessed the impact of a mindful movement program, known as Movimento Biologico (MB), on participants psychological well-being (PWB), positive mental health (PMH), sense of coherence (SOC), and interoceptive awareness.

**Methods:**

MB program was conducted for students attending the bachelor’s degree in Kinesiology and Sport Sciences of University of Perugia over 8 weeks (from October 16 to November 27, 2022). Participants were requested to fill in four questionnaires before and after the MB program: (1) 18-item PWB scale; (2) 9-item PMH scale; (3) 13-item SOC scale; (4) 32-item scale for Multidimensional Assessment of Interoceptive Awareness (MAIA). Wilcoxon signed-rank tests were used to assess changes, with significance set at *p* < 0.05.

**Results:**

Thirty-eight students (mean age 21.2, 60.5% male) participated. Several MAIA subscales, including noticing (*p* = 0.003), attention management (*p* = 0.002), emotional awareness (*p* = 0.007), self-regulation (*p* < 0.001), body listening (*p* = 0.001), and trusting (*p* = 0.001), showed significant improvements. PMH increased significantly (*p* = 0.015), and there was a significant enhancement in the autonomy subscale of PWB (*p* = 0.036). SOC and overall PWB also improved, though not significantly.

**Conclusion:**

The MB program significantly improved participants’ positive mental health and interoceptive awareness. This likely resulted from better recognition and management of positive physiological sensations, a stronger link between physical sensations and emotions, enhanced confidence in one’s body, and increased autonomy.

## Introduction

Young adults, also known as emerging adults, are people between the late teens and twenties (1) who experience a critical period for their individual’s development, with lasting implications for their health, well-being, and economic stability. Contrary to popular belief, they can be surprisingly unhealthy, exhibiting an even worse health profile than thirty-year-olds (2). Therefore, it is crucial to support them in successfully transitioning from adolescence to adulthood (2, 3) addressing their mental health needs, and promoting both mental and physical health and well-being.

Mental health refers to a state of emotional, psychological, and social well-being in which individuals can effectively cope with the demands of everyday life (4).

It encompasses various dimensions, including cognitive functioning, emotional regulation, a sense of connection to others and interpersonal relationships, and the ability to cope with adversity (5).

Mental health and psychological well-being are fundamental to individuals’ overall functioning, quality of life, and ability to adapt to the challenges they encounter. The World Health Organization (WHO) recognizes the essential role of psychological well-being and mental health promotion, describing it as “a key function for ensuring healthy lives and promoting well-being at all ages.” This highlights the need for greater attention from public health in promoting mental health from a salutogenic perspective (6).

There is international evidence (7, 8) regarding feasible and effective interventions to promote mental health.

Psychoeducational and cognitive-behavioral interventions, relaxation programs, mindfulness and meditative/mindful movement (MM) practices are among the interventions found to be effective in improving mental well-being in the young adult population (9). Mindfulness is defined as intentionally directing attention to present-moment experiences in a non-judgmental way (10), with an orientation to curiosity, openness, acceptance and non-reactivity (11, 12). Mindfulness-based interventions have evolved over time and currently also include programs centered on MM (13, 14).

MM interventions (15) are practices of mindful movement that aim to dissolve the mind–body separation promoting instead their integration. They rely on theories (16, 17) that emphasize how the experience of oneself in the world as conscious being involves a complex interaction of the brain, body, and environment, and the seamless integration of interoceptive, proprioceptive (including vestibular), kinesthetic, tactile, and spatial information (18–20). These practices are indeed animated by the desire to overcome the historical Cartesian dualism of mind and body separation (21) in search of the ontological unit of an articulated multiplicity, as human being is (22).

Mind–body dualism is actually not biologically plausible (23) because the mind is not to be regarded either a disembodied internal representation of the external world, nor as a system of brain modules, neural symbols, and algorithms. On the contrary, an embodied mind manifests and integrates the current state of the entire organism as it interacts with its environment (24).

Furthermore, movement of the body can reveal effects on the structure and function of the brain (25–28). Interactions between brain, mind, body and behavior can be used to promote health, enhancing well-being, mood, sleep, cognition (29), attention, learning, emotion regulation (30) and positively impacting stress reduction and self-regulation (31). MM practices, such as yoga, qigong, and tai chi, incorporate the purposeful regulation of movement and coordination between the breath, mind, and body (32) and are based on mental presence and a meditative attitude. Movimento Biologico® (MB) is a MM intervention characterized by the integration of formal meditation techniques (body awareness, breathing awareness, walking meditation, loving-kindness meditation) and informal meditation (during daily activities). It encompasses psycho-education on stress and emotional management, dialogic practices, breathing practices, free expressive movements, motor games, experiential anatomy practices, motor problem solving exercises, self-knowledge diaries, vocal practices and many other forms of teaching-learning mediated by movement (33, 34) harmonized with each other through the filter of body pedagogics and embodied learning. (35, 36).

MM interventions have already been shown to alleviate symptoms of various clinical conditions (37, 38) and induce measurable changes in physiological markers of stress (39, 40), cognitive functions (41, 42), and sensory motor acuity (43). Changes in several psycological outcomes were also assessed in the general adult or older adult population with positive results shown in respect to quality of life (44), depressive symptoms (44, 45), anxiety (45), and sleep quality (44, 46). Recent studies have also examined such practices in adolescents (47, 48) to investigate their impact on cognitive functions and young adults (49–51). The latter evaluated specific aspects, such as depression, stress, quality of life and affective state without considering the broad concept behind psychological well-being.

It emerges from the above that research on MM is relatively less developed in respect to that on conventional meditation and exercise and physical activity (52) and this could be due to the multifaceted nature of MM interventions, which typically involve sequences of specific movements, the particular use of breathing and the modulation of attention (53). Furthermore, despite the current interest in “mind–body medicine,” the Cartesian paradigm persists, as does the continuing gap between MM and traditional medical and psychiatric theory (54).

Considering MM characteristics, it is reasonable to expect that MM can positively impact psychological, emotional, and social well-being (55, 56), namely the “Positive Mental Health” (PMH). From a salutogenic perspective, better PMH can positively impact the capacity to utilize resources, i.e., the sense of coherence (SOC), a cornerstone of the salutogenic model that relates to individuals’ ability to manage life events (57). The connection between PMH, SOC, and salutogenesis is implicit in the definition of PMH as expressed by the WHO (58), which states that PMH represents the foundation of individuals’ well-being and effective functioning. On the other hand, SOC expresses individuals’ ability to manage and give meaning to internal and external stimuli from their environment over the course of life, valuing their capacity to use resources to cope with difficulties and maintain better physical and mental health (59, 60). Indeed, both salutogenesis (61) and PMH focus on the construction of those “positive qualities” that embody the positive vision of health on one hand and positive psychology on the other (62, 63).

Based on this foundation, the present study aims to evaluate the short-term impact of a MM program known as Movimento Biologico® (MB) on these domains in a young adult population.

## Materials and methods

### Study design and population

We conducted an experimental pilot study without a control group. The study population included subjects aged 18 to 26 years old, attending the 2nd and 3rd year of the bachelor’s degree in Kinesiology and Sport Sciences at the University of Perugia. Individuals with pre-existing psychological disorders or those currently involved in competitive sports, mindfulness activities, or MM practices were excluded. The number of participants was set at 40 for logistical issues and the enrollment was voluntary, with applications collected during the second half of September 2022 through the project’s designated contact person. Applications were considered on a first-come, first-served basis. Students were informed of the possibility to enroll in the program through the website of the Degree in Kinesiology and Sport Sciences, as well as students mailing list and WhatsApp group. The training program was advertised after the project received the approval from the Local Ethics Committee.

### Intervention: the mindful movement program Movimento Biologico® (MB)

The MB program was led by an expert with a background in Kinesiology and Sports Sciences with 17 years of experience in the field of MM, neuroscience and meditation. The program lasted 8 weeks (from October 16th to November 27th, 2022) and consisted of 8 intensive sessions of 4 or 8 h, carried out in groups associated with activities to be carried out individually as homework.

It was based on a set of activities that allowed for a more meaningful and evolutionary contact with the substantial dimension of the person, namely corporeality, and through it, with the unique, creative, and spiritual depth of the personality. The program included theoretical and practical sessions, as well as self-managed activities to be carried out at home. The sessions were conducted with the whole group while the “homework” practices were carried out independently by each participant and without direct supervision.

The group sessions included moments of psycho-education, meditation and contemplation practices (such as body scan, breathing awareness, walking meditation, loving-kindness meditation), individual motor practices (such as kinesphere, floorwork, juggling, and experiential anatomy), motor games in partnering, dialogic mindfulness practices, vocal practices, creation of drawings, diaries for self-knowledge, motor problem solving exercises and other teaching-learning tools. The group activities took place in the gyms of the Degree Course in Kinesiology and Sports Sciences.

The homework practices were facilitated using supporting audio or video files and paper materials (such as diaries) that were provided to participants during the group sessions. It was not possible to systematically monitor the homework, therefore, we relied on the participants’ ethics and adherence to the program. The total duration of the program was 48 h, with a maximum of 8 h of allowed absence that was set to minimize the risk of not being able to reliably assess the impact of the program for a loss of potential achievable benefits.

Table 1 provides a summary of the intervention while the detailed program is included in Supplementary material.

**Table 1:** MB program sessions and activities.

Session	Duration	Theoretical contents of education in conscious embodiment	Body–mind practices	Homework
1.	8 H	Reflection on expectations, anticipation, and motivations | Exploration of conceptual horizons: awareness, embodiment, self-care, attention, transformative “technologies,” pillars of practice	An Mo & Do In (self-massage movements) | Self-analysis and breath re-education | Kinesphere [use of an imaginary sphere for movement stimulation] (KS) | Body Scan	Body Scan + KS
2.	4 H	\	Sitting Meditation [breath awareness (BA)] | Guided experiential anatomy | Statue, Snail, Jellyfish | Free exploration in floorwork (floor movements) (FEF) | Dialogic practice	BA + FEF
3.	4 H	Obstacles to practice and “antidotes”	Dialogic practice | Review of practices from the previous session (BA, FEF, KS)	BA + FEF + KS
4.	8 H	Modes of “doing” vs. “being” | Relationship between body, emotions, and thoughts ABC model (antecedents, beliefs, consequences) | Autopilot and living in the head | Decentralization	Sitting Meditation (personal analysis and motivation) | Body-thoughts-emotions exercise based on mindfulness | Breathing Space	BA + Pleasant Events Diary + Breathing Space
5.	4 H	Stressors and stress | Reaction vs. response	Dialogic practice | Balance games with a partner | Juggling	BA + Problem Solving Foot Block + Unpleasant Events Diary + Breathing Space
6.	4 H	Salutogenesis (promotion of health) | General resources of resilience | Sense of coherence	Sitting Meditation (personal resources practice) | Walking Meditation	BA + Walking Meditation
7. + 8.	16 H	Opening to experience: relationship, problem-solving, expressiveness, vocalization, empathy	Sitting Meditation (Open Monitoring) (Loving Kindness, Compassion) (Equanimity) - Trust Practice | Dialogic Practice | Auditory and Vocal Practices | Floorball | Matrix Practice | Steals the handkerchief | Juggling | Balance Practice | Orchestra Conductor | Guiding the other’s body with your touch | Mimesis | Moving the Body at a Distance | Dancing each other’s dance | Pass the cup of water as a group	Self-Management with Personalized Biographical Practices

### Study procedures and data management

During the enrollment, a structured interview was conducted by the research staff to collect socio-demographic data (date and month of birth, gender, residential address, citizenship, educational qualification) and clinical-anamnestic data (self-perceived health, pre-existing medical conditions, depressed mood in the last 2 weeks, medication intake, lifestyle habits such as smoking, alcohol consumption and physical activity, sports involvement). Body mass index (BMI) was calculated considering self-reported weight and height.

The questions regarding smoking habits addressed three main aspects: the current smoking status of the person, the quantity of cigarettes smoked and the duration of smoking habit, and the length of time since they quit smoking for former smokers. The questions about alcohol habits focused on the total number of alcoholic units consumed within a 30-day period, and on consumption of 6 or more alcoholic units on a single occasion (binge drinking). An alcoholic unit is made up of a can of beer (330 mL) or a glass of wine (125 mL) or a shot of liquor (40 mL).

The questions regarding physical activity included inquiries about whether the individual had moderate/intense physical activity in the last 30 days and its weekly duration. Furthermore, in order to identify physical active people responders were requested to say if they had at least 30 min of moderate physical activity on at least 5 days a week and/or 20 min of intense physical activity on at least 3 days a week.

### Measures

Additionally, participants were requested to complete four questionnaires before and after the MB program:

1. An 18-item questionnaire, validated in Italian, to assess the Psychological Well-Being (PWB) in terms of six dimensions, namely self-acceptance, positive relationships with others, autonomy, environmental mastery, purpose in life, personal growth. Each item of this questionnaire is rated on a 6-point Likert scale ranging from 1 to 6, where 1 represents “Completely disagree” and 6 represents “Completely agree.” Higher scores indicate higher levels of well-being. The Cronbach’s alpha (α) of the questionnaire is 0.52 for self-acceptance, 0.56 for positive relationships with others, 0.37 for autonomy, 0.49 for environmental mastery, 0.33 for purpose in life, and 0.40 for personal growth (64).
2. A 9-item questionnaire, validated in Italian, to measure Positive Mental Health (PMH). Each item of this questionnaire is rated on a 4-point Likert scale ranging from 0 (strongly disagree) to 3 (strongly agree). Higher scores indicate higher PMH. The Cronbach’s alpha is 0.93 (65).
3. A 13-item questionnaire, validated in Italian, to evaluate the Sense of Coherence (SOC) and its three underpinning dimensions, namely the cognitive (Comprehensibility), the instrumental or behavioral (Manageability) and the motivational dimension (Meaningfulness). Each item of this questionnaire is rated on a 7-point Likert scale ranging from 1 (never) to 7 (always). Higher scores indicate greater levels of SOC. The Cronbach’s alpha is 0.83 (66).

A 32-item questionnaire, validated in Italian, known as the Multidimensional Assessment of Interoceptive Awareness (MAIA), to assess the perception and interoceptive awareness of the body and its dimensions (noticing, not distracting, not worrying, attention regulation, emotional awareness, self-regulation, body listening, and trusting). Each item of this questionnaire is rated on a 6-point Likert scale from 0 to 5 with ‘0’ indicating ‘Never’ and ‘5’ indicating ‘Always’. Higher scores equate to more awareness of bodily sensation. Cronbach’s alpha vary among the subscales: noticing (*α* = 0.69), Not-Distracting (*α* = 0.66), Not-Worrying (*α* = 0.67), Attention Regulation (*α* = 0.87), Emotional Awareness (*α* = 0.82), Self-Regulation (*α* = 0.83), Body Listening (*α* = 0.82) and Trusting (*α* = 0.79) (67). The total score as well subscales scores were calculated pre- and post-intervention.

### Statistical analysis

A descriptive analysis was performed through absolute and relative frequencies and means (± standard deviation) as opportune.

The Wilcoxon signed-rank test was executed to determine whether MB program produced a significant difference in post-intervention scores. The choice of a non-parametric test was led by the small sample size. A *p*-value of less than 0.05 was considered statistically significant.

Effect sizes (ES) were calculated dividing the mean difference between post and pre intervention by the standard deviation of the difference. ES were categorized as small (ES = 0.2), medium (ES = 0.5), and large (ES ≥ 0.8), according to the benchmarks proposed by Cohen (68).

A secondary analysis was also performed excluding participants who exceeded the 8-h absence limit.

All analyses were conducted using STATA 14.0 (Stata Corp ltd, TX).

## Results

Thirty-eight students (mean age 21.2 years; range 20–25; SD 1.2; 60.5% males) attended the MB program. Sociodemographic and clinical anamnestic data are presented in Table 2.

**Table 2:** Population’s sociodemographic and clinical anamnestic data.

Variable		*N*.
Sex	Male	23 (60.5%)
Female	15 (39.5%)
Age	Mean (±SD)	21.2 (± 1.2)
BMI	- Underweight (16–18.49)	3 (7.9%)
	- Healthy weight (18.5–24.99)	27 (71%)
	- Overweight (25–29.99)	6 (15.8%)
	- Obesity class 1 (30–34.99)	2 (5.3%)
Self-perceived health	- Very good	10 (26.3%)
	- Good	20 (52.6%)
	- Average	5 (13.2%)
	- Fragile	3 (7.9%)
	- Very fragile	0 (0%)
Depression mood	- Yes	10 (26.3%)
	*Mean of days with depression mood*	*5.2* (*SD 2.7*)
	- No	28 (73.7%)
Smoke	- Smoker	17 (44.7%)
	*Mean N° of cigarettes smoked weekly (±SD)*	*38.9 (±43.9)*
	*Months of smoking, mean (±SD)*	*44.5* (*±25.7*)
	Ex- smoker	9 (23.7%)
	*≥ Six months*	*6* (*66.7%*)
	*< Six months*	*2* (*22.2%*)
	*Unknown*	*1* (*11.1%*)
	Non- smoker	12 (31.6%)
Alcohol (at least 1 alcohol unit) during the past 30 days	- Yes	27 (71.1%)
	*Mean N° of alcohol unit during the past 30 days* (*± SD*)	*1.1 (±0.7)*
	*Binge drinking*	*3* (*11.1%*)
	- No	11 (28.9%)
Physical activity (Moderate-intense physical activity during the past 30 days)	- Yes	32 (84.2%)
	*Mean moderate-intense physical activity* (*min/week*) (*±SD*)	*337.7* (*±270.2*)
	- No	6 (15.8%)
Physical active	- Yes	29 (76.3%)
	- No	9 (23.7%)

Six students (15.8%) did not reach the expected amount of attendance hours (hours of absence: min 8h30min, max 16 h, mean 10h52min).

In Table 3, pre- and post-intervention scores are shown.

**Table 3:** Pre and post MB program scores.

		PRE			POST				
		Median	Interquartile range	SD	Median	Interquartile range	SD	*p*	ES
MAIA	**Noticing**	3.75	3–4	0.84	4.25	3.75–4.50	0.63	**0.003**	0.53
	**Not-distracting**	2	1.67–2.67	0.81	2.33	1.67–2.67	0.70	0.689	0.07
	**Not worrying (37)**	2.67	2–3.33	1.02	2.67	2–3.67	1.06	0.934	0.02
	**Attention regulation**	2.86	2.29–3.29	0.79	3.57	2.86–3.86	0.78	**0.002**	0.60
	**Emotional awareness**	4	3.2–4.4	0.93	4.1	3.8–4.6	0.49	**0.007**	0.50
	**Self-regulation**	2.75	2.25–3.25	1.00	3.5	2.75–4.25	1.01	**<0.001**	0.74
	**Body-listening**	2.67	1.67–3.33	1.01	3.67	2.67–4	1.07	**0.001**	0.67
	**Trusting**	3.67	2.67–4	1.18	4	3.33–5	0.82	**0.001**	0.60
PWB	**Self-acceptance (37)**	12	10–13	2.46	12	11–14	2.11	0.286	0.24
	**Positive relations with others**	15	12–17	3.02	14.5	12–17	2.56	0.796	0.05
	**Autonomy**	14	10–15	3.17	14.5	13–15	2.66	**0.036**	0.43
	**Environmental mastery (36)**	12	11–14	2.36	13	10–14	2.49	0.769	0.08
	**Purpose in life (36)**	12	11–13	2.14	13	10–13	1.76	0.223	0.18
	**Personal growth**	16	15–18	2.16	16	14–17	2.02	0.491	−0.11
	**Total (34)**	84.5	73.5–88.5	11.97	84	75.5–90	9.18	0.387	0.18
PMH	**Total (37)**	18.0	15–21	4.87	19	16–22	4.06	**0.015**	0.46
SOC	**Comprehensibility (36)**	20.5	17–22.5	5.28	21.5	19–25	4.52	0.161	0.25
	**Manageability**	17	13–20	4.83	15.5	12–18	4.22	0.153	−0.25
	**Meaningfulness**	20.5	16–23	4.62	21	17–24	3.73	0.271	0.21

Significant *p*-values are reported in bold.

The majority of the MAIA subscales, including noticing (*p* = 0.003), attention regulation (*p* = 0.002), emotional awareness (*p* = 0.007), self-regulation (*p* < 0.001), body listening (*p* = 0.001), and trusting (*p* = 0.001), improved significantly after the MB program. A significant increase also occurred in PMH score (*p* = 0.015).

On the other hand, except for the autonomy subscale of PWB (*p* = 0.036), neither PWB nor SOC scores improved significantly.

Medium ESs were observed for all the scores that showed significant changes with higher values for MAIA subscales. The secondary analysis conducted excluding participants who exceeded the absence limit did not show any notable difference in the results (Table 4).

**Table 4:** Pre and post MB program scores in subjects who had not exceeded the limit of hours of absence.

		PRE			POST				
		Median	Interquartile range	SD	Median	Interquartile range	SD	*p*	ES
MAIA	**Noticing**	3.88	3.13–4.25	0.73	4.25	3.75–4.50	0.50	**0.009**	0.51
	**Not-distracting**	2	1.67–2.83	0.82	2.33	1.67–2.67	0.74	0.881	0.04
	**Not worrying (31)**	2.67	1.67.3.33	1.02	2.67	1.83–3.33	1.06	0.617	0.09
	**Attention regulation**	2.86	2.29–3.21	0.76	3.57	2.86–3.93	0.72	**0.001**	0.70
	**Emotional awareness**	4	3.3–4.4	0.87	4.1	3.8–4.6	0.46	**0.008**	0.51
	**Self-regulation**	2.75	2.25–3.13	0.96	3.5	2.75–4.25	0.98	**<0.001**	0.84
	**Body-listening**	2.83	2.17–3.5	0.97	3.67	2.5–4	1.08	**0.004**	0.61
	**Trusting**	3.67	2.5–4	1.26	4	3.33–5	0.83	**0.002**	0.63
PWB	**Self-acceptance**	11.5	10–14	2.61	12	11–14	2.18	0.552	0.20
	**Positive relations with others**	14.5	12–16	3.07	15	12–17	2.66	0.247	0.28
	**Autonomy**	14	10.5–15.5	3.30	15	13–16	2.43	**0.017**	0.54
	**Environmental mastery (30)**	12	10–14	2.50	13	10–15	2.45	0.498	0.22
	**Purpose in life (31)**	11	10–13	2.13	12.5	10–13	1.80	0.135	0.28
	**Personal growth**	16	14.5–18	2.29	16	14–17.5	2.06	0.663	0.06
	**Total (29)**	84	72–88	12.69	84	75–91	9.60	0.108	0.31
PMH	**Total (31)**	18	14–21	4.81	19	16–23	4.22	**0.013**	0.51
SOC	**Comprehensibility (30)**	21	16–23	5.37	21.5	19–25.5	4.75	0.108	0.31
	**Manageability**	16	13–19.5	4.96	15	11.5–18.5	4.40	0.167	−0.24
	**Meaningfulness**	20	16–23	4.58	21	18–24	3.80	0.110	0.31

Significant *p*-values are reported in bold.

## Discussion

The findings of our study provide evidence that the MB program had positive effects on interoceptive awareness and psychological mental health among young adults.

We observed significant changes in the most of MAIA subscales. Participants demonstrated improved abilities to recognize body sensations, manage attention, become more emotionally aware, engage in self-regulation, listen to their bodies, and establish trust in their bodily experiences. These findings are consistent with previous research that has found that MM practices, such as yoga and tai chi, improve interoceptive awareness (69–71) and could be interesting in the light of the known relationship between interoceptive awareness and emotional regulation (72, 73).

Furthermore, the MB program resulted in a significant increase in psychological mental health, as assessed by the PMH scale. This finding suggests that the training could have enhanced the participants’ general well-being and mental functioning. In this respect MM activities have already been shown to reduce stress, anxiety, and depressive symptoms, while also fostering positive emotions and psychological resilience (74–76)_._ The participants’ engagement in MB program exercises might have provided opportunities for self-expression, stress reduction, and emotional regulation, leading to improved PMH.

As far as psychological well-being is concerned, only the PWB autonomy subscale showed significant changes. The autonomy subscale measures an individual’s perception of having control over their own actions, choices, and behaviors. It reflects the extent to which a person feels independent, self-directed, and able to make decisions aligned with their own values and interests (77). Autonomy is a central aspect in the developmental process of emerging adults who face evolutionary challenges such as starting work, leaving the parental home, and engaging in stable and lasting relational experiences. Higher levels of autonomy during this phase of life are viewed as “being self-determined and independent; being able to resist social pressures in thinking and acting; being capable of self-regulating one’s behavior; and evaluating oneself based on personal standards” (78). Therefore, the results shown by our study could be interesting as autonomy can have a positive impact on health and well-being (78, 79).

Regarding the SOC, the results of our study did not show significant improvements. The SOC refers to an individual’s perception of the world as comprehensible, manageable, and meaningful, and it has been associated with better mental health and well-being (80). The lack of significant improvements in SOC may be attributed to several factors. One possibility is that the relatively short duration of the program was not enough to impact the SOC. It is possible that a longer intervention period may have allowed for a more comprehensive exploration and cultivation of participants’ sense of comprehensibility, manageability, and meaningfulness but this needs to be addressed in further studies.

Additionally, it should be noted that the activities included in the intervention program were mostly directed to target the dimensions of the PWB and interoceptive awareness. Nonetheless the program also included a specific activity on salutogenesis and was expected to potentially improved SOC because it enhanced “internal” resistance resources, namely physical, psychic, relational, emotional, mental, cognitive, intellectual and spiritual potential of the individual.

To the best of our knowledge our study is rather unique in terms of type of intervention proposed and broadness of outcomes assessed. In fact, other studies on the same target population, namely young adults, have mostly investigated mindfulness-based interventions that gave priority to the mindfulness practices. Systematic reviews of studies evaluating mindfulness-based interventions in university students have shown improvements in respect to stress and mindfulness (81–83)^,^ mental health and well-being (81–83) but also coping capacities (82).

The unprecedented integration that MB proposes among the multiple dimensions of motor skills (sensory, perceptive, expressive, symbolic, communicative, relational, playful, creative, cognitive, spiritual) is meant to constitute a pragmatic “bridge” of interaction between body, mind, and emotions in line with the emerging mind–body medicine. In this context, the body, mind, and emotions are regarded as working in unity (84). Furthermore, MB is characterized as a tool for enriching “body awareness,” a term frequently used (67, 85–87) to define a mental function characterized by a keen sensitivity to bodily signals, which leads to conscious identification of states deriving from subtle bodily reactions to internal and external conditions (88). In this respect MB focuses on the quality of movement, on “how” movements are experienced in relation to space, time and energy (89). The “embodiment” of experience constitutes a potential pillar for a deeper and more fruitful bodily awareness and could be a tool for improving the functional quality of specific neural processes, allowing the cultivation of an attentional ability that may be transferred to multiple contexts and areas (90, 91).

This is a rather “new” approach since the evaluations done by clinicians and researchers is still driven by anatomical, biomechanical and physiological parameters, despite the large spectrum of psychological, social and humanistic dimensions that the movement contains (92).

It is important to note some limitations of this research. Firstly, a control group was not foreseen, and the study population was relatively small and selected from a specific university context. These limits prevent to assess the efficacy of the program in a robust way and to generalize the results, but it should be observed that the purpose of this study was exploratory.

Another limit is due to the fact that the impact of the program was evaluated just in a short-term horizon and only in healthy students not affected by any psychological disorder. In particular, the exclusion of students with pre-existing psychological disorders was reasoned by the evidence (93) that psychological disorders might influence sensory perception and subjective evaluation of environmental stimuli and physical activities and, subsequently, the impact of the intervention. It should also be considered that, because of the small sample size, stratified analyses were inconclusive (results not shown). Nevertheless, in future studies it would be important to address if socio-demographic variables, such as sex, as well as personal lifestyles could influence the impact of the program. Finally, we need to consider that the low Cronbach’s alpha value of the domains of the PWB questionnaire could impair results in terms of their reproducibility but it should be also considered that the 18-item questionnaire is widely used in research because of its validity.

Our study has also some strengths, including the use of validated tools for evaluating the program impact and the good level of engagement of participants that was demonstrated by the absence of dropouts and by the high level of questionnaire completion. Furthermore, the positive results issued by our pilot study provide the basis for planning further studies also including a control group and a longer follow up.

Our results provide preliminary evidence of the potential of MB as a mean to promote well-being and physical and mental health during the critical period of young emerging adulthood. The positive preliminary results showed could be attributed to a positive physiological sensation recognition and management, a deeper connection between physical sensations and emotional states, and a greater perception of one’s own body. Further research with different study designs and larger and diverse study populations are envisaged to confirm and expand upon these preliminary findings and to explore the mechanisms underlying the observed effects.

## Data availability statement

The raw data supporting the conclusions of this article will be made available by the authors, without undue reservation.

## Ethics statement

The studies involving humans were approved by the Comitato Etico Regionale dell’Umbria. The studies were conducted in accordance with the local legislation and institutional requirements. The participants provided their written informed consent to participate in this study.

## Author contributions

SSP: Writing – review & editing. MC: Writing – review & editing. FI: Writing – review & editing. LB: Writing – review & editing. GP: Writing – review & editing. RC: Writing – original draft. CG: Writing – original draft. CL: Writing – original draft. AC: Writing – review & editing. GS: Writing – review & editing. CM: Writing – review & editing. AB: Writing – review & editing. CdW: Writing – original draft.

## References

[1] Arnett JJ. Emerging adulthood: what is it, and what is it good for? Child Dev Perspect. (2007) 1:68–73. doi: 10.1111/j.1750-8606.2007.00016.x

[2] Stroud C Walker LR Davis M Irwin CE Jr. Investing in the health and well-being of young adults. J. Adoles. Health. (2015) 56:127–9. doi: 10.1016/j.jadohealth.2014.11.012 25620297

[3] Committee on Improving the Health, Safety, and Well-Being of Young Adults; Board on Children, Youth, and Families; Institute of Medicine; National Research Council In: Bonnie RJ Stroud C Breiner H, editors. Investing in the health and well-being of young adults. Washington, DC: National Academies Press (2015) 25855847

[4] World Health Organization. Promoting mental health: Concepts, emerging evidence, practice: Summary report. Geneva: World Health Organization (2004).

[5] O'Reilly M Lester JN. Examining mental health through social constructionism: The language of mental health. Cham, Switzerland: Palgrave Macmillan. (2017). doi: 10.1007/978-3-319-60095-6

[6] Keyes CL Dhingra SS Simoes EJ. Change in level of positive mental health as a predictor of future risk of mental illness. Am J Public Health. (2010) 100:2366–71. doi: 10.2105/AJPH.2010.192245, PMID: 20966364 PMC2978199

[7] Barry MM Clarke AM Petersen I Jenkins R. Implementing mental health promotion. 2nd ed. Cham, Switzerland: Springer Nature (2019).

[8] Herrman H Jané-Llopis E. The status of mental health promotion. Public Health Rev. (2012) 34:1–21. doi: 10.1007/bf0339167426236074

[9] Conley CS Durlak JA Kirsch AC. A meta-analysis of universal mental health prevention programs for higher education students. Prev Sci. (2015) 16:487–507. doi: 10.1007/s11121-015-0543-1, PMID: 25744536

[10] Kabat-Zinn J. Full catastrophe living: Using the wisdom of your body and mind to face stress, pain and illness. New York, NY: Delacorte (1990).

[11] Baer RA. Mindfulness training as a clinical intervention: a conceptual and empirical review. Clin Psychol Sci Pract. (2003) 10:125.

[12] Bishop SR Lau M Shapiro S Carlson L Anderson ND Carmody J. Mindfulness: a proposed operational definition. Clin Psychol Sci Pract. (2004) 11:230–41. doi: 10.1093/clipsy.bph077

[13] Crane RS Brewer J Feldman C Kabat-Zinn J Santorelli S Williams JMG. What defines mindfulness-based programs? The warp and the weft. Psychol Med. (2017) 47:990–9. doi: 10.1017/S0033291716003317 28031068

[14] Kabat-Zinn J. Mindfulness-based interventions in context: past, present, and future. Clin Psychol Sci Pract. (2003) 10:144–56.

[15] Larkey L Jahnke R Etnier J Gonzalez J. Meditative movement as a category of exercise: implications for research. J Phys Act Health. (2009) 6:230–8. doi: 10.1123/jpah.6.2.230, PMID: 19420401

[16] Barsalou LW. Grounded cognition. Annu Rev Psychol. (2008) 59:617–45. doi: 10.1146/annurev.psych.59.103006.093639 17705682

[17] Krueger DW. Integrating body self & psychological self. Creating a new story in psychoanalysis and psychotherapy. 2nd ed. New York, NY: Routledge Press (2002).

[18] Ehrsson HH. The experimental induction of out-of-body experiences. Science. (2007) 317:1048. doi: 10.1126/science.1142175 17717177

[19] Haselager WFG Broens MC Gonzalez MEQ. The importance of sensing one's movement in the world for the sense of personal identity. Rivista internazionale di Filosofia e Psicologia. (2012) 3, 1–11. doi: 10.4453/rifp.2012.0001

[20] Ionta S Gassert R Blanke O. Multi-sensory and sensorimotor foundation of bodily self-consciousness - an interdisciplinary approach. Front Psychol. (2011) 2:383. doi: 10.3389/fpsyg.2011.00383, PMID: 22207860 PMC3245631

[21] Damasio A. Descartes' error: emotion, reason, and the human brain.10th ed. New York: Penguin Books (2005).

[22] Brencio F. World, time and anxiety. Heidegger's Existential Analytic Psychiatry Folia medica. (2014) 56:297–304. doi: 10.1515/folmed-2015-0011 26444361

[23] Van Bael K Ball M Scarfo J Suleyman E. Assessment of the mind-body connection: preliminary psychometric evidence for a new self-report questionnaire. BMC Psychol. (2023) 11:309. doi: 10.1186/s40359-023-01302-3, PMID: 37803484 PMC10557351

[24] Fuchs T. The circularity of the embodied mind. Front Psychol. (2020) 11:1707. doi: 10.3389/fpsyg.2020.01707, PMID: 32903365 PMC7434866

[25] Han YMY Chan MMY Choi CXT Law MCH Ahorsu DK Tsang HWH. The neurobiological effects of mind-body exercise: a systematic review and meta-analysis of neuroimaging studies. Sci Rep. (2023) 13:10948. doi: 10.1038/s41598-023-37309-4, PMID: 37415072 PMC10326064

[26] Linchevski I Maimon A Golland Y Zeharia N Amedi A Levit-Binnun N. Integrating mind and body: investigating differential activation of nodes of the default mode network. Restor Neurol Neurosci. (2023) 41:115–27. doi: 10.3233/RNN-231334, PMID: 37742669 PMC10741374

[27] Liu J Tao J Xia R Li M Huang M Li S. Mind-body exercise modulates locus Coeruleus and ventral tegmental area functional connectivity in individuals with mild cognitive impairment. Front Aging Neurosci. (2021) 13:646807. doi: 10.3389/fnagi.2021.646807, PMID: 34194314 PMC8236862

[28] Zhang X Zong B Zhao W Li L. Effects of mind-body exercise on brain structure and function: a systematic review on MRI studies. Brain Sci. (2021) 11:205. doi: 10.3390/brainsci11020205, PMID: 33562412 PMC7915202

[29] Laird KT Paholpak P Roman M Rahi B Lavretsky H. Mind-body therapies for late-life mental and cognitive health. Curr Psychiatry Rep. (2018) 20:2. doi: 10.1007/s11920-018-0864-4, PMID: 29372339

[30] Muehsam D Lutgendorf S Mills PJ Rickhi B Chevalier G Bat N. The embodied mind: a review on functional genomic and neurological correlates of mind-body therapies. Neurosci Biobehav Rev. (2017) 73:165–81. doi: 10.1016/j.neubiorev.2016.12.027, PMID: 28017838

[31] Esch T Stefano GB. The BERN framework of mind-body medicine: integrating self-care, health promotion, resilience, and applied neuroscience. Front Integr Neurosci. (2022) 16:913573. doi: 10.3389/fnint.2022.913573, PMID: 35910341 PMC9330052

[32] Rice LC Deronda AC Kiran S Seidl K Brown K Rosch KS. Mindful movement intervention applied to at risk Urban School children for improving motor, cognitive, and emotional-behavioral regulation. Mindfulness. (2023) 14:637–47. doi: 10.1007/s12671-022-02063-7, PMID: 36744072 PMC9887233

[33] Spaccapanico Proietti S. Umanizzare il movimento. Roma: Armando Editore (2021).

[34] Spaccapanico Proietti S. Movimento e consapevolezza: le pratiche di mindfulness e mindful movement nella promozione della salute. Roma: Armando Editore (2022).

[35] Kelly M Ellaway R Scherpbier A King N Dornan T. Body pedagogics: embodied learning for the health professions. Med Educ. (2019) 53:967–77. doi: 10.1111/medu.13916, PMID: 31216603

[36] van der Kamp J Withagen R Orth D. On the education about/of radical embodied cognition. Front Psychol. (2019) 10:2378. doi: 10.3389/fpsyg.2019.02378 31749732 PMC6848271

[37] Jahnke R Larkey L Rogers C Etnier J Lin F. A comprehensive review of health benefits of qigong and tai chi. AJHP. (2010) 24:e1–e25. doi: 10.4278/ajhp.081013-LIT-248, PMID: 20594090 PMC3085832

[38] Wren AA Wright MA Carson JW Keefe FJ. Yoga for persistent pain: new findings and directions for an ancient practice. Pain. (2011) 152:477–80. doi: 10.1016/j.pain.2010.11.017, PMID: 21247696 PMC3040510

[39] Lee MS Kang CW Lim HJ Lee MS. Effects of qi-training on anxiety and plasma concentrations of cortisol, ACTH, and aldosterone: a randomized placebo-controlled pilot study. Stress Health. (2004) 20:243–8. doi: 10.1002/smi.1023

[40] West J Otte C Geher K Johnson J Mohr DC. Effects of hatha yoga and African dance on perceived stress, affect, and salivary cortisol. Annals Behav Med. (2004) 28:114–8. doi: 10.1207/s15324796abm2802_6, PMID: 15454358

[41] Manjunath NK Telles S. Improved performance in the tower of London test following yoga. Indian J Physiol Pharmacol. (2001) 45:351–4. https://pubmed.ncbi.nlm.nih.gov/11881575/. PMID: 11881575

[42] Silva LM Cignolini A Warren R Budden S Skowron-Gooch A. Improvement in sensory impairment and social interaction in young children with autism following treatment with an original qigong massage methodology. Am J Chin Med. (2007) 35:393–406. doi: 10.1142/S0192415X07004916, PMID: 17597498

[43] Kerr CE Shaw JR Wasserman RH Chen VW Kanojia A Bayer T. Tactile acuity in experienced tai chi practitioners: evidence for use dependent plasticity as an effect of sensory-attentional training. Exp Brain Res. (2008) 188:317–22. doi: 10.1007/s00221-008-1409-6, PMID: 18512052 PMC2795804

[44] Weber M Schnorr T Morat M Morat T Donath L. Effects of mind-body interventions involving meditative movements on quality of life, depressive symptoms, fear of falling and sleep quality in older adults: a systematic review with Meta-analysis. Int J Environ Res Public Health. (2020) 17:6556. doi: 10.3390/ijerph17186556, PMID: 32916879 PMC7559727

[45] Wang F Lee EK Wu T Benson H Fricchione G Wang W. The effects of tai chi on depression, anxiety, and psychological well-being: a systematic review and meta-analysis. Int J Behav Med. (2014) 21:605–17. doi: 10.1007/s12529-013-9351-9 24078491

[46] Wang F Eun-Kyoung Lee O Feng F Vitiello MV Wang W Benson H. The effect of meditative movement on sleep quality: a systematic review. Sleep Med Rev. (2016) 30:43–52. doi: 10.1016/j.smrv.2015.12.001, PMID: 26802824

[47] Kang H An SC Kim NO Sung M Kang Y Lee US. Meditative movement affects working memory related to neural activity in adolescents: a randomized controlled trial. Front Psychol. (2020) 11:931. doi: 10.3389/fpsyg.2020.00931, PMID: 32477223 PMC7236766

[48] Marson F Fano A Pellegrino M Pesce C Glicksohn J Ben-Soussan TD. Age-related differential effects of school-based sitting and movement meditation on creativity and spatial cognition: a pilot study. Children. (2021) 8:583. doi: 10.3390/children8070583 34356562 PMC8303844

[49] Edwards MK Loprinzi PD. Affective responses to acute bouts of aerobic exercise, mindfulness meditation, and combinations of exercise and meditation: a randomized controlled intervention. Psychol Rep. (2019) 122:465–84. doi: 10.1177/0033294118755099, PMID: 29368545

[50] Lavadera P Millon EM Shors TJ. MAP train my brain: meditation combined with aerobic exercise reduces stress and rumination while enhancing quality of life in medical students. J Alt Comp Med. (2020) 26:418–23. doi: 10.1089/acm.2019.0281, PMID: 32310686

[51] Pascoe MC Parker AG. Physical activity and exercise as a universal depression prevention in young people: a narrative review. Early Interv Psychiatry. (2019) 13:733–9. doi: 10.1111/eip.12737, PMID: 30302925

[52] Kabir RS Yang HJ. Editorial: meditative movement for mental and physical health. Front Psychol. (2023) 14:1238633. doi: 10.3389/fpsyg.2023.1238633, PMID: 37469902 PMC10352907

[53] Wayne PM Kaptchuk TJ. Challenges inherent to t'ai chi research: part I—t'ai chi as a complex multicomponent intervention. J Altern Complement Med. (2008) 14:95–102. doi: 10.1089/acm.2007.7170A, PMID: 18199021

[54] Leder D. A tale of two bodies: the Cartesian corpse and the lived body In: The body in medical thought and practice. Dordrecht: Springer (1992). 17–35.

[55] Keyes CL Shmotkin D Ryff CD. Optimizing well-being: the empirical encounter of two traditions. J Pers Soc Psychol. (2002) 82:1007–22. doi: 10.1037/0022-3514.82.6.1007, PMID: 12051575

[56] Keyes CL. Promoting and protecting mental health as flourishing: a complementary strategy for improving national mental health. Am Psychol. (2007) 62:95–108. doi: 10.1037/0003-066X.62.2.95, PMID: 17324035

[57] Eriksson M. The sense of coherence in the Salutogenic model of health In: Mittelmark MB Sagy S Eriksson M Baue GF Pelikan JM Lindström B, editors. The handbook of Salutogenesis. Cham, Switzerland: Springer (2016). 91–6.

[58] World Health Organization. Quinta Conferencia Internacional de Promoción de la Salud. Promoción de la salud: hacia una mayor equidad. Ciudad de México: World Health Organization (2000).

[59] Eriksson M Lindström B. Antonovsky's sense of coherence scale and its relation with quality of life: a systematic review. J Epidemiol Community Health. (2007) 61:938–44. doi: 10.1136/jech.2006.056028, PMID: 17933950 PMC2465600

[60] Gustavsson-Lilius M Julkunen J Keskivaara P Lipsanen J Hietanen P. Predictors of distress in cancer patients and their partners: the role of optimism in the sense of coherence construct. Psychol Health. (2012) 27:178–95. doi: 10.1080/08870446.2010.484064, PMID: 21391129

[61] Antonovsky A. Health. Stress and Coping, San Francisco: Jossey-Bass (1979).

[62] Joseph S Sagy S. Positive psychology and its relation to Salutogenesis In: Mittelmark MB Bauer GF Vaandrager L Pelikan JM Sagy S Eriksson M, editors. The handbook of Salutogenesis. 2nd ed. Cham: Springer (2022) 36122004

[63] Togari T Yamazaki Y Takayama TS Yamaki CK Nakayama K. Follow-up study on the effects of sense of coherence on well-being after two years in Japanese university undergraduate students. Personal Individ Differ. (2008) 44:1335–47. doi: 10.1016/j.paid.2007.12.002

[64] Ryff CD Keyes CL. The structure of psychological well-being revisited. J Pers Soc Psychol. (1995) 69:719–27. doi: 10.1037//0022-3514.69.4.719 7473027

[65] Lukat J Margraf J Lutz R van der Veld WM Becker ES. Psychometric properties of the positive mental health scale (PMH-scale). BMC Psychol. (2016) 4:8. doi: 10.1186/s40359-016-0111-x, PMID: 26865173 PMC4748628

[66] Sardu C Mereu A Sotgiu A Andrissi L Jacobson MK Contu P. Antonovsky's sense of coherence scale: cultural validation of Soc questionnaire and socio-demographic patterns in an Italian population. Clin Pract Epidemiol Mental Health. (2012) 8:1–6. doi: 10.2174/1745017901208010001, PMID: 22371812 PMC3282882

[67] Mehling WE Price C Daubenmier JJ Acree M Bartmess E Stewart A. The multidimensional assessment of interoceptive awareness (MAIA). PLoS One. (2012) 7:e48230. doi: 10.1371/journal.pone.0048230, PMID: 23133619 PMC3486814

[68] Cohen J. Statistical power analysis for the behavioral sciences. New York, NY: Routledge Academic (1988).

[69] Fiori F Aglioti SM David N. Interactions between body and social awareness in yoga. J. Alt. Comp. Med. (2017) 23:227–33. doi: 10.1089/acm.2016.0169 27854495

[70] Mehling WE Wrubel J Daubenmier JJ Price CJ Kerr CE Silow T. Body awareness: a phenomenological inquiry into the common ground of mind-body therapies. PEHM. (2011) 6:6. doi: 10.1186/1747-5341-6-6, PMID: 21473781 PMC3096919

[71] Parkinson TD Smith SD. A cross-sectional analysis of yoga experience on variables associated with psychological well-being. Front Psychol. (2023) 13:999130. doi: 10.3389/fpsyg.2022.999130, PMID: 36743606 PMC9889934

[72] Kever A Pollatos O Vermeulen N Grynberg D. Interoceptive sensitivity facilitates both antecedent-and response-focused emotion regulation strategies. Personal Individ Differ. (2015) 87:20–3. doi: 10.1016/j.paid.2015.07.014

[73] Pollatos O Schandry R. Emotional processing and emotional memory are modulated by interoceptive awareness. Cognit Emot. (2008) 22:272–87. doi: 10.1080/02699930701357535

[74] Antonini Philippe R Schwab L Biasutti M. Effects of physical activity and mindfulness on resilience and depression during the first wave of COVID-19 pandemic. Front Psychol. (2021) 12:700742. doi: 10.3389/fpsyg.2021.700742, PMID: 34393936 PMC8360111

[75] Cramer H Lauche R Langhorst J Dobos G. Yoga for depression: a systematic review and meta-analysis. Depress Anxiety. (2013) 30:1068–83. doi: 10.1002/da.22166 23922209

[76] Pascoe MC Thompson DR Ski CF. Yoga, mindfulness-based stress reduction and stress-related physiological measures: a meta-analysis. Psychoneuroendocrinology. (2017) 86:152–68. doi: 10.1016/j.psyneuen.2017.08.008, PMID: 28963884

[77] Delbosc A Vella-Brodrick D. The role of transport in supporting the autonomy of young adults. Transport Res F: Traffic Psychol Behav. (2015) 33:97–105. doi: 10.1016/j.trf.2015.03.011

[78] Ryff CD. Happiness is everything, or is it? Explorations on the meaning of psychological well-being. J Pers Soc Psychol. (1989) 57:1069–81. doi: 10.1037/0022-3514.57.6.1069

[79] Chatzisarantis NL Ada EN Ahmadi M Caltabiano N Wang D Thogersen-Ntoumani C. Differential effects of perceptions of equal, favourable and unfavourable autonomy support on educational and well-being outcomes. Contemp Educ Psychol. (2019) 58:33–43. doi: 10.1016/j.cedpsych.2019.02.002

[80] Galletta M Cherchi M Cocco A Lai G Manca V Pau M. Sense of coherence and physical health-related quality of life in Italian chronic patients: the mediating role of the mental component. BMJ Open. (2019) 9:e030001. doi: 10.1136/bmjopen-2019-030001, PMID: 31530606 PMC6756344

[81] Dawson AF Brown WW Anderson J Datta B Donald JN Hong K. Mindfulness-based interventions for university students: a systematic review and Meta-analysis of randomised controlled trials. Appl Psychol Health Well Being. (2020) 12:384–410. doi: 10.1111/aphw.12188, PMID: 31743957

[82] Kaisti I Kulmala P Hintsanen M Hurtig T Repo S Paunio T. The effects of mindfulness-based interventions in medical students: a systematic review. Adv Health Sci Educ. (2023) 29:245–71. doi: 10.1007/s10459-023-10231-0 PMC10927869 37227541

[83] O’Driscoll M Byrne S Mc Gillicuddy A Lambert S Sahm LJ. The effects of mindfulness-based interventions for health and social care undergraduate students–a systematic review of the literature. Psychol Health Med. (2017) 22:851–65. doi: 10.1080/13548506.2017.1280178, PMID: 28103700

[84] Schulz S Cysarz D Seifert G. Mind-body medicine and its impacts on psychological networks, quality of life, and health. Front Integr Neurosci. (2023) 17:1188638. doi: 10.3389/fnint.2023.1188638, PMID: 37082088 PMC10111009

[85] Gyllensten AL Skär L Miller M Gard G. Embodied identity—a deeper understanding of body awareness. Physiother Theory Pract. (2010) 26:439–46. doi: 10.3109/09593980903422956, PMID: 20649495

[86] Ginzburg K Tsur N Barak-Nahum A Defrin R. Body awareness: differentiating between sensitivity to and monitoring of bodily signals. J Behav Med. (2014) 37:564–75. doi: 10.1007/s10865-013-9514-9, PMID: 23633239

[87] Mattsson M. Body awareness: Applications in physiotherapy. Umeå, Sweden: Umeå Universitet (1998).

[88] Dropsy J. Le corps bien accordé: un exercice invisible. Paris: Epi (1984).

[89] Skjaerven LH Kristoffersen K Gard G. An eye for movement quality: a phenomenological study of movement quality reflecting a group of physiotherapists' understanding of the phenomenon. Physiother Theory Pract. (2008) 24:13–27. doi: 10.1080/01460860701378042, PMID: 18300105

[90] Clark D Schumann F Mostofsky SH. Mindful movement and skilled attention. Front Hum Neurosci. (2015) 9:297. doi: 10.3389/fnhum.2015.00297 26190986 PMC4484342

[91] Langer EJ. Mindful learning. Curr Dir Psychol Sci. (2000) 9:220–3. doi: 10.1111/1467-8721.00099

[92] Gibson B. Rehabilitation: A post-critical approach. Boca Raton, FL: CRC Press (2016).

[93] Elkjær E Mikkelsen MB Michalak J Mennin DS O'Toole MS. Motor alterations in depression and anxiety disorders: a systematic review and meta-analysis. J Affect Disord. (2022). doi: 10.1016/j.jad.2022.08.060, PMID: 36037990

