# Supplementary material for: The role of a mindful movement-based program (Movimento Biologico) in health promotion: results of a pre-post intervention study
Source: Front Public Health. 2024 Jun 11;12:1372660. doi: 10.3389/fpubh.2024.1372660 (PMC11196965; doi:10.3389/fpubh.2024.1372660)
Supplement: Supplementary file 1 [file Table_1.docx]

Supplementary Material

**Movimento Biologico Program**

From October 16 to November 27, 2022

**Background and key elements of the program:**

Movimento Biologico (MB) is characterized by a multidimensional approach to movement that combines its multiple dimensions (sensory, perceptive, expressive, symbolic, communicative, relational, playful, creative, cognitive, spiritual, ...).

To do this the program is characterized by the integration of formal meditation techniques and informal meditation, psycho-education on stress and emotional management, dialogic practices, breathing practices, free expressive movements, motor games, experiential anatomy practices, motor problem solving exercises, self -knowledge diaries, vocal practices and many other forms of teaching-learning mediated by movement harmonized with each other through the filter of body pedagogics and embodied learning.

A particularly characterizing element of MB is the constant observation of "*how I do what I do*": the awareness of "how" I move, "how" I feel, "how" I relate, "how" I look, "how" I touch, " how" I get excited, etc., directing attention to the potential for movement that goes in the direction of health, through sensitivity to the spectrum of movement nuances in response to multiple internal, external and relational conditions.

An approach, the one that MB tries to make participants learn, based on the embodiment of experience as all learning is rooted within personal experience.

A learning not based on "*learning things through one's own movement*" (notions, information, data - according to a rationalist perspective), nor merely on "*learning through one's own movement*" (exercises, activities aimed at a purpose - according to a functionalist perspective), but rather on “*learning by being inside learning*”, “*learning by embodying one's own movement*” (Dewey, 1934,

Arnold, 1973, Skjaerven *et al.*, 2019). Sensitivity and attention are interconnected.

Attention can be considered similar to "conscious observation" that encompasses a wide range of sensory, perceptual and sentimental experiences.

*Consciously observe the movement.*

*Observe yourself in motion. Move the observation. Observing the moving of the observation.*

*Moving, allowing the dialogic relationships between the inside and outside of the body we are to emerge.*

*Move by carefully observing the spectrum of sensitivity of the experience.*

*Move observing the Self. Allow yourself to be moved by the Self.*

*Being in contact with the Self and observing yourself from the inside.*

**Program description**

Each session of the program is described in this way:

- Duration
- General presentation of the session objectives
- Detailed description of contents; for contents designed to specifically impact the psycho-physical skills of the participant (for example, exercises, psycho-education spaces, homework) the expected impact area of the intervention is indicated in brackets () and italics
- Homework

**Session 1: 8 hours**

**Presentation**

The first session was designed to introduce the program with its characterizing elements and form a "learning pact" with the participants.

It was considered essential, respecting the indications of the Mindfulness Based Programs, to clarify to the group the horizon to be explored and experienced, its ontological roots, the expectations, the attitude to be cultivated and to make each one responsible towards a virtuous commitment to be guaranteed throughout the entire process.

The semantic aspects of meditation were exposed in detail, as well as its ability to be a privileged "means" to become "researchers" of mental dynamics, "explorers" of knowledge of the functioning mechanisms of our inner world. Returning to Foucault, meditation as a "technology of the Self" (Kelly, 2013).

The seven pillars (foundations) of the practice, the components and types of meditation and its phases were illustrated.

The objective was to offer participants detailed and clear knowledge on what meditation is (and what it is not), what its historical roots are, its functions, how it fits into the context of work practices on the Self, how to extricate oneself from preliminary difficulties, how to begin to “turn inward” in a consciously aware way.

The initial techniques of bodily sensorial awareness, respiratory education and the motor practice of the Kinesphere were subsequently proposed.

**Contents**

- **Welcome and introduction to the program**

The introduction was deliberately short and not markedly detailed to avoid pre-conditioning the group.

- **Description of the “learning agreement”**

A few minutes were dedicated to sharing the substantial points for the success of the journey:

- - privacy and decorum,
  - respect for generative silence,
  - attention and care for one's own needs and those of others,
  - listening to the other without interrupting him or giving advice,
  - commitment to cultivating one's daily practice for at least thirty minutes a day,
  - possibility of relating to the instructor for doubts, curiosities, clarifications, insights.
- **Education on the horizon to aim for**

The teacher explained to the group the direction to turn to through semantic, philosophical and scientific references.

It was explained how, wanting to summarize the meaning of the program, it is driven by the objective of experiencing techniques, exercises, tools of awareness. A psycho-corporeal instance characterized by «being fully present, present to the senses, to the intentionality of contact» which «serves to keep us constantly updated on ourselves. A continuous process, accessible at any time, and not an exclusive or sporadic enlightenment that can only be achieved at particular times or in particular conditions».

It is through awareness, according to Kabat-Zinn, that we can bring out a wide range of self-regulation skills through the development and refinement of specific internal resources, which contribute to «developing healthy and positive behaviors and resilience on a psychological level and emotional» (Haruki, 1996).

- **Opening practice: "guided" reflection on one's personal motivations (*attention regulation, emotional awareness, body listening, purpose in life, autonomy*)**

After a brief moment of meditation guided by three deep breaths, I ask myself: “*Why am I here? What is my intention? What do I expect from this journey*?”.

The participants were accompanied, in their silent reflection, by some orientation and meaning inputs.

They were invited to deeply observe, without prejudice or judgment, their own attitude through exemplary dichotomies.

One of these was that of the "pirate" and the "pilgrim", the "hunter" and the "cultivator".

I ask myself, lightly and openly: "*Am I here to learn techniques, contents, practices (pirate, hunter) or am I here to open myself up to the experience as it is, giving myself the opportunity to abandon certainties (pilgrim, farmer)?*".

Another suggestion was that of "hedonic" or "eudaimonic" intentionality (Ryff, 2021).

I ask myself, with vivid and sincere discernment: “*Am I here for an exquisitely individual purpose, for my singular happiness, to 'take' something from the experience (see pirate, hunter), or am I here to cultivate a personal elevation to be put at the service of others, to 'offer' myself to the experience (see pilgrim, farmer)?*”

I don't judge the answers. I don't care about a label. I simply become aware of what I encounter and observe it, with discernment but without judgment.

The activity of discernment is crucial.

Discernment produces knowledge. Discernment is a knowledge that has to do with separation.

- **An-Mo | Do-In (*noticing, body listening, not distracting, attention regulation, emotional awareness*)**

The teacher led the group in a self-massage practice conducted in a standing position, aimed at increasing body skin sensitization through touch (Zhu *et al.,* 2016, Liu *et al.*, 2021).

The practice is characterized by rubbing, vibrations, pressures, initially light and then deeper on all the main parts of the body, starting from the face and reaching the feet.

The intention of the practitioners is oriented towards increasing their attentional focus on bodily listening, on the emotions of the present moment and on the associated flows of thoughts.

- **Breath Self-Analysis (*noticing, attention regulation, body listening, autonomy*)**

Through self-observation, participants were invited to evaluate the ability of their breath to distribute itself in the various parts of their trunk (abdomen, lower chest, upper chest) and in the various directions (anterior, posterior, lateral, superior, inferior).

A numerical rating scale (0-5) was used through which each participant could assign a score to the various districts. The scoring was based on the subjective perception of respiratory intensity in a given district.

The self-observation was accompanied by the teacher's guidance which marked the transition from observation of one district to another.

- **Breathing self-education (*noticing, attention regulation, body listening, self-regulation, trusting, not distracting, autonomy, self-acceptance, positive mental health*)**

Some self-stretching exercises have been proposed for the front, rear and lateral parts of the trunk, associated with deep breathing to be directed towards the specific area being stretched.

The objective was to re-educate the participants to be able to voluntarily direct the respiratory volume in the various districts of the trunk so as to be able to make the respiratory process more global and fluid (Tavoian and Craighead, 2023).

- **Body Scan Practice (*noticing, attention regulation, emotional awareness, body listening, not distracting, positive mental health*)**

In a position lying comfortably on the ground, the participants were invited to preliminarily pay attention to their breathing, to its natural flow, observing the sensations of breathing moment by moment.

Multiple suggestions were offered: observing the gentle movement of expansion and retraction of the abdomen and chest, feeling the freshness of the air entering during inhalation and the heat of the air exiting during exhalation, following the tactile perceptions of air along the airways (nostrils, throat, bronchi, lungs).

Breath should be savored, observed, not controlled or judged.

The attention is totally present to the breath, to the present moment of the breath.

When a thought, a memory, a mental image, a physical sensation, a sound or an emotion distracts attention from breathing, we consciously notice it and gently bring attention back to breathing.

From awareness of breathing, we move on to Body Scan, to the conscious observation of body sensations (Gan *et al.*, 2022, Anālayo, 2020, Dambrun *et al.*, 2019). The participants were guided within this practice to observe, district after district, all the main anatomical areas of their body: from the ends of the toes to the apex of the skull.

The attention rests, following the instructor's guidance, on the part of the body to be observed. It is not important that the sensation emerges clearly (heat, heaviness, lightness, tingling, tension, etc.); what matters is that the attention is directed to that part, is based on that part.

Also in this case, as previously, when a thought, a memory, a mental image, a physical sensation in a different point, a sound or an emotion distracts attention from the part of the body on which we are, with awareness we notice it and gently bring our attention back to the breath and then to the part of the body we were observing.

- **Education in the knowledge of Mindfulness and Contemplative Practices: Part 1 (*autonomy, personal growth, environmental mastery, purpose in life*)**

The teacher offered an educational space on the ontological and teleological aspects of Mindfulness and, more generally, Contemplative Practices.

Starting from the concept of awareness, this semantic construct was applied to "taking care".

As seen previously, by cultivating awareness we access ways of virtuous use of our internal resources and develop those self-regulation skills capable of igniting healthy and positive behaviors and resilience on a psychological and emotional level.

All this is an example of a "care" attitude.

Cultivating awareness means "taking care".

It is interesting to delve deeper into the etymology of "cure". It derives from the Greek *epimèleia* which has close affinities with Melete, the first of the nine Muses of Olympus.

Daughter of Mnemosyne and Zeus, she was the muse of thought and meditation. Melete literally means "meditate" and "contemplation" in Greek.

“Taking care” is intimately connected to the meditative attitude, to the meditative act.

But “take care” of what? Whose?

About how we do what we do, whatever we're doing.

Observe ways of acting and being. Recognize the "how" of "things" through direct examination of experience.

Pay attention to the “how” of the experience.

Taking Edmund Husserl (Butler, 2016, Overgaard, 2008), seeing the "how" compared to seeing the "what" (visual judgement).

Seeing the "how" of the experience, of one's own experience: the giving, the manifestation, the emergence of the experience.

A "taking care" that becomes *epimèleia heautoù*, the "taking care of oneself" of Socratic memory.

But how to “take care of yourself”?

And, even before that, what does "taking care" really mean?

There could be many answers to this question. What is considered interesting and useful to explore is linked to attention.

Attention is a cognitive process that "manifests itself" in terms of the ability to channel mental resources onto specific aspects of reality at certain moments.

It is the mechanism capable of selecting incoming information based on its biological and/or psychological relevance for the individual.

Quoting Merleau-Ponty (Merleau-Ponty, 1962, 1964, Merleau-Ponty and Edie, 1964), «the structure of attention is presence». Being there comes before thinking about it.

Attention is constitutive, it is creative: «it actively constructs a new object that explains and thematizes what was previously offered only as an indeterminate horizon».

In other words, it is not the experience that "captures" attention, it is attention that generates the experience.

Vital energy is not something that exists independently of attention, but is produced by attention.

Blowing on the wings of this fascinating perspective, caring means, in essence, paying attention.

Pay attention to the “how” of the experience.

Pay attention to the "how" of being which, following Francisco Varela's interpretation, coincides with *experience*.

Paying attention transforms the mental field, creates a new way for consciousness to be present to its objects.

Carlo Rovelli's (Rovelli 2014, 2018, 2020) suggestion is illuminating: "the mere fact of observing (carefully) is sufficient to modify reality".

Even more illuminating, perhaps, is Merleau-Ponty's suggestion: «I am not a series of psychic acts, nor for that matter a central ego that brings them together in synthetic unity, but a single experience inseparable from itself [...], I am a field". A field of attention.

Developing a theory of attention and embodying its application principles to one's existence constitutes, at least from the writer's point of view, the essence of any path of self-care, of self-transformation.

Go beyond the current form of things, transcend the current form of oneself, cultivate one's incessant need for mutation.

How we pay attention defines the experience. Generate the experience. The experience changes.

And what is the "art of changing" if not spirituality, just as Foucault defined it.

A dimension that can be reached through physical, theoretical, and psychic modalities entirely aimed at the transformation of the self.

A transformation of the vision of the world which is a true metamorphosis of the personality.

The subject is transfigured by the return effect of the truth that he experiences on himself.

Where to start from on this path of mutation, of transformation based on attention?

Definitely from the body. Whether you choose to "honor" the tradition of Contemplative Practices or whether you choose to "embrace" the contemporary perspective of neuroscience.

“The body is the zero point of all orientations.”

A body, as seen in the previous chapters, which is not a mere organism but a place of feeling and, therefore, of being.

The group was accompanied, by these founding pillars, towards the knowledge of the embodied mind, the philosophical-physiological construct that states how the world and mind are mutually constituted on the basis of the bodily experiences we have of the world.

- **Kinesphere Practice (*noticing, attention regulation, emotional awareness, body listening, self-regulation, autonomy, environmental mastery, positive mental health*)**

It is the first markedly motor practice. After having experienced the body scan practices in a lying position, the participants were offered the opportunity to come into contact with their own corporeity in a more dynamic way, so as to be able to recognize differences and similarities between the multiple practice opportunities.

The Kinesphere is a practice adapted from Laban's choreological intuitions (Block, 1998), characterized by a specific setting: the subject, in an upright position with bare feet, first comes into deep contact with his own body. Observe the breech pressures on the ground, the sense of lightness or heaviness, of opening or closing, of expansion or condensation of the body and its parts. Then he is invited to generate free movements in space, having the only constraint of not being able to move his feet from the ground compared to his original position. The goal of these free, exploratory movements is to embody the physical space that the body can actually occupy in movement.

The image of the Kinesphere is offered: “*imagine being inside a sphere and, through your movements, imagine touching the internal walls of this sphere*”.

The group was guided, after this first initial experience, to a series of further "in-depth studies".

For example, the suggestion of the brush was used: "*imagine having a brush attached to the tip of the middle finger of your right hand and being able to color as much of the surface of the Kinesphere in which you are immersed; while 'painting' pay attention to the sensations of the body, observe the quality of the movement, recognize if thoughts, images, intuitions, memories, emotions emerge*".

Then, subsequently, the object of suggestion was changed: “*now imagine replacing the brush attached to the tip of the middle finger of the right hand with a pencil with a thin tip and observe how the quality of the experience changes; what happens to the movement, how does it change the speed, the fluidity, the sensation of density of the arm, the heaviness or lightness of the hand, the emotional state?*".

Then, yet another new suggestion: “*now imagine that the brush is attached to the top of the head and that you still want to color the internal walls of the Kinesphere: how the movement now changes, is it more natural or unnatural, is it free or rigid, is it serene or restless?*”.

Then, further stimuli: “*relax your arm along your body and observe if there are differences with the other; try not to have a judgmental attitude but simply limit yourself to observing the quality of one and the other: notice the perception of length, width, weight, density, volume, temperature, touch and any other parameter that you can grasp. Now spontaneously raise your right arm and observe the quality of this experience, do it a few times until you can clearly understand what you feel. Now, instead, imagine that what lifts your right arm is a thread attached to the back of your wrist; the intention to move the arm arises from the wrist; more, by a thread attached to the back of the wrist: how does the experience change? How much does your arm weigh now? Not the biological, physical, mathematical weight of the arm. What is the 'biographical' weight of the arm now? What is the density of the arm now? Is it different from before? It's not important that you label it, you just need to recognize if there is a difference.*

*What if now, instead of there being a thread that lifts the arm, the intention of movement were born from the palm of a hand that supports, from bottom to top, the right wrist, from below. Imagine a hand that softly accompanies your wrist upwards. How is the experience now? What quality does your movement have? What is alive in you?*”

These are just some of the examples, suggestions, intentions, which were offered to the group to acquire a vivid and embodied awareness of how there are multiple - potentially infinite - ways to produce the "same" movement.

We continued on other parts of the body, with other "images" capable of eliciting a "mixed" attentional stimulus (internal-external): I listen to the body, I observe its qualities, its sensations (internal focus) while, however, I am busy to co-observe the fluidity of my brush stroke, to explore the full breadth of the Kinesphere, to modify the kinetic intentionality between "being pulled", "being supported", "expanding", "condensing" (external focus).

Concluding the practice, we ended with a phase of free exploration in which each participant was invited to move freely within the Kinesphere, spontaneously generating intentions and movement impulses without allowing themselves to be guided by the teacher's instructions any longer.

Everyone was invited to continue to be totally present to the experience, totally absorbed in observing the quality of the experience, moment by moment, without falling into the temptation to choreograph, perform, compare or judge their own movement compared to that of the other or to an ideal mental model.

The purpose of the practice of the Kinesphere is to offer the opportunity to recognize, being totally absorbed by the bodily experience, the differences, the pluralities, the sensorial, cognitive, symbolic and emotional nuances of the experience itself.

- **Education in the knowledge of Mindfulness and Contemplative Practices: Part 2 (*autonomy, personal growth, environmental mastery, purpose in life*)**

After the experience of the Body Scan and the Kinesphere, the educational framework of Mindfulness and Contemplative Practices was completed.

We started from meditation, from its etymologies: *bhāvanā* (Sanskrit) which can be translated as "cultivate, develop something" and *gom* (Tibetan) which can be translated as "become familiar with".

Cultivate, develop what? Familiarize yourself with what? Ourselves. Our mind. Our sensations, our emotions, our internal world.

Meditation is essentially stopping to bring attention from outside to inside as Seneca already suggested with his *rĕvertĕre in sē*.

Being "scientists of ourselves": observing, collecting data, knowing and understanding the mechanisms of our inner world.

In this, as seen previously, meditation can be considered one of the "technologies of the Self".

Those technologies which according to Foucault (Foucault, 1988, 2016) «allow individuals to carry out, with their own means or with the help of others, a certain number of operations on their own body and soul – from thoughts, to behavior, to the way of being – and to realize thus a transformation of oneself with the aim of achieving a state characterized by happiness, purity, wisdom, perfection or immortality".

It is interesting to note how historically the image of a "technique" was already present long before Foucault.

For example, Plato, in Alcibiades I, inserts a *téchne* to take care of one's *psyché*, the center of basic vital functions and intellectual and emotional life.

Why is there a need?

Because we risk not taking care of it by thinking we are taking care of it (self-deception, illusion, ignorance, not seeing how things are); and because we are exposed to innumerable causes of discomfort and suffering, to what the Latins will call perturbationes animi.

The *psyché*, prisoner of images and projections that it does not recognize as such and whose true origin it ignores, can free itself through a process of arduous ascent, a process of education, discovery and knowledge of what is around, but also of its own possibilities.

The techniques of the *psyché* are techniques of self-liberation, of ascent, through knowledge of oneself and of reality, which allow one to become capable of distinguishing what is real and what is only apparently real.

Also in Patañjali's Yoga Sūtra the importance of a "method (*yoga*) which aims at definitively stopping the whirling plexus of cognitions (*cittavṛtti*)" is underlined.

*Citta*, the counterpart of the Greek *psyché*, is a complex term: «Intellect, egoity, and mind are brought together into a single all-pervasive cognitive faculty called awareness (*citta*)» (Larson, 1987) which, however, we could consider what we currently qualify as mind.

The whirlwind pace of mental processes [(vortex of cognitions (*cittavṛtti*)] is the existential and atavistic "problem" of Man of all time.

A method (*yoga*) is needed to put an end to this “problem” (*cittavṛtti*).

With a parallel to the Plato mentioned previously, we could say that knowing the whirlwind pace of mental processes coincides with knowing oneself and being able to derive many advantages from it.

The term *yoga* defines the homologue of the previously mentioned *téchne*.

In summary, *yoga* could be associated with the "method", the "technique" which aims to stop the whirlwind pace of mental processes.

Meditating is, essentially, knowing and governing the swirling flows of the mind and is capable of inducing being in the world in a different way.

The purpose of this broad historiographical framework on meditation was to make the group aware of the roots of the practices, of their profound meaning and of the openings and suggestions that this collective "consciousness" that has thrived over the centuries can elicit.

This educational part concluded with the description of the components, types, phases and pillars of a mindfulness practice.

Drawing on the tradition of Tibetan Buddhism, the parallelism of "*ingredients*" and "*cake*" was used to explain the different characteristics of a practice.

The components of the practice are the counterpart of the "*ingredients*" while the types of meditation are the counterpart of the "*cake*".

The "*ingredients*" of a meditative practice can be summarized in two great qualities or instances: sustained attention and awareness.

In relation to the "dosage" of these *ingredients*, a "*cake*" can be obtained which we will qualify as "calm abiding" (*Śamatha*) or as "clear vision" (*Vipassanā*).

In "calm abiding" meditations the "*ingredient*" sustained attention is prevalent. These are, therefore, so-called "attentive" meditations in which the focus is on practicing recognizing distraction and returning to the object of meditation (breath, body, sounds, etc.).

In "clear vision" meditations the "*ingredient*" awareness is prevalent. In this case, these are so-called "analytical" or "reflective" meditations characterized by a greater orientation towards the profound analysis of experience (analysis that leads to the understanding of concepts, mental ideas, interdependence, emptiness, etc.).

It is clear that this division tends to be brutally simplistic and reductive. The aim is rather to create the basis for a clear understanding of how to navigate the practice. The epistemological framework is decidedly more complex and articulated.

The "phases" of a formal practice were subsequently explained, i.e. how to set up a meditation session.

The four phases are:

1. Preparation – this is the phase that brings together the introductory moments in which we prepare for the subsequent phases. We ensure that the body is in a comfortable, dignified, stable but not rigid condition, to be able to support the practice and, subsequently, we enter a state of greater "centering", generally paying attention to the breathing with the aim of calming the mind and reduce the tendency to distraction.

2. Motivation – is the phase in which a high, broad, altruistic intent is generated that supports the practice. For example, one can generate the strong intention to strive to develop a more virtuous sense of Self so as to be of benefit to all sentient beings.

3. Effective Meditation – it is the central phase, the "heart" of the practice and varies depending on what you have decided to take as the "object" of meditation (breath, body, movement, etc.).

4. Dedication - it is the "closing" moment of the practice and is characterized by the desire to dedicate the positive energy generated by the joyful effort of meditation, and the benefits that will derive from it, to those who are suffering, to those who have no causes and conditions to be able to practice, to all the people who made our practice possible (our teachers who gave us the opportunity to learn to read, write, understand, know, understand; our family members who, perhaps, take care of the house while we are practicing, etc.) and, more generally, to all sentient beings.

Homework

- **Body Scan Practice** (20 minutes a day)
- **Kinesphere Practice** (20 minutes a day)

An audio guide file was provided with which the participants could carry out the two daily practices.

**Session 2: 4 hours**

**Presentation**

The second session focused on breathing awareness meditation experiences and individual and relational conscious movement and dialogic practices.

**Contents**

- **Sitting Meditation: Breathing Awareness (*noticing, attention regulation, emotional awareness, body listening, not distracting, self-acceptance*)**

First of all, as a first knowledge, the "seven-point meditation posture" was presented - and experienced.

*1. The legs*

Sit, if possible, with your legs crossed in the vajra, or full lotus, position, where each foot rests, with the sole facing upwards, on the thigh of the other leg. This position is difficult to achieve, but by training your body, you can gradually achieve it. This position offers the best support to the body and mind. However, it is not essential.

An alternative position is half lotus, where one foot is on the floor under the opposite leg and the other foot is on top of the other thigh.

A third alternative is to simply sit cross-legged, with both feet flat on the floor under opposite thighs.

Sitting on a firm cushion that raises your buttocks higher than your knees can be a great help in keeping your spine straight. It also helps you sit longer without your legs or feet falling asleep, and avoids annoying tingling.

If it is not possible to sit on the floor on a cushion, a low meditation bench can be used.

It's also fine to meditate while sitting in a chair. The most important thing is to find a suitable position in which you are comfortable.

*2. The Arms*

Keep your hands relaxed in your lap, with the back of your right hand resting softly on the palm of your left. Thumbs touch each other.

In this way the hands will look like a drop or a flame. Hold them about five centimeters below the navel.

Alternatively, rest the concavity of your palms to wrap around the convexity of your knees.

Relax your shoulders and arms. The arms should be slightly detached from the body, so as to guarantee free expansion of the trunk volumes during breathing.

*3. The Back*

The back is the most important part.

It must be extended, relaxed but well erect, as if the vertebrae were blocks stacked effortlessly in a column. This helps maintain clarity and focus of the mind during meditation.

The position of the legs can help enormously to easily keep the back erect; often, the higher the pillow under the pelvis and the lower the knees are, the easier it becomes to keep the back upright.

*4. The Eyes*

When starting out, it's easier to concentrate with your eyes completely closed. That's fine, however, as you gain experience in meditation, it is advisable to learn to keep your eyes slightly half-closed to let some light filter in, looking downwards, without focusing on anything in particular. Keeping your eyes completely closed can induce drowsiness, sleep, or dreamlike images, all of which hinder clear meditation.

*5. The mouth*

The jaw should be relaxed, the teeth not clenched and the lips touching softly.

*6. The Tongue*

The tongue should rest naturally on the roof of the mouth, with the tip touching the root of the upper incisors. This reduces the flow of saliva and therefore the need to swallow. These spontaneous activities of the body, becoming distractions, can be an obstacle to the deepening of meditation.

*7. The Head*

Your head should be slightly tilted forward, so that you can naturally direct your gaze towards the floor in front of you. If you hold your chin too high, you may have problems with distraction, the mind wandering. Holding your head too low, however, can lead to mental numbness or drowsiness.

Subsequently, we entered into the practice of Breath Awareness (Schöne *et al.*, 2018, Anālayo, 2019, Mo *et al.*, 2021).

Participants were invited to bring their attention to their breathing, to the sensations produced in the body by natural breathing, moment by moment.

As in the preliminary phases of the Body Scan, multiple suggestions were offered: observing the gentle movement of expansion and retraction of the abdomen and chest, feeling the freshness of the air entering during inhalation and the heat of the air exiting during exhalation, follow the tactile sensations of the air along the airways (nostrils, throat, bronchi, lungs).

No will to control the breathing is exercised.

The breath is simply observed, not controlled or judged.

The attention is totally present to the breath, to the present moment of the breath. When a thought, a memory, a mental image, a physical sensation, a sound or an emotion distracts attention from breathing, we consciously notice it and gently bring attention back to breathing.

- **Guided Experiential Anatomy (*noticing, attention regulation, emotional awareness, body listening, not distracting, self-acceptance, positive mental health*)**

The objective of the guided Experiential Anatomy practice (Berland, 2018, Brown *et al.*, 2011, Glaser, 2015) was to make the group experiment, with a further sensorimotor modality, the concept of "variability", "alternative", "multimodality": the same motor response, the same gesture, the same simple and elementary movement, can be produced, manifested, processed and decoded in multiple ways.

By way of example, lying supine on the floor, each participant was invited to observe the sensations resulting from the contact of the thoracic region with the floor during head movement.

*What do I feel and "how" does my chest feel when I rotate my head to the right without "imprinting" a particular intention?*

*How does that same feeling change if I rotate my head with the intention of "exploiting" the convexity of the back of my head? If I imagine that my neck is a sphere on which I can softly and fluidly roll.*

*How does the feeling change if I try to "spread" the back ribs, the shoulder blades and the vertebrae on the ground, abandoning all the anterior tensions that "hold me" towards the ceiling?*

*Can I recognize the anatomical parts of the chest in this "smearing"?*

*If I suspend the rotation movement of the head and stop to observe the breathing, can I recognize the expansion of the lungs and expand the chest posteriorly?*

*Can I, precisely through the observation of breathing, recognize the harmony of the tissue micro-adjustments that allow this rhythmic pulsation?*

*Can I, by following the tactile sensation of the air inside the airways, visualize the "perimeter" of my lungs?*

*Can I, by observing the lower ribs, lateral to the projection of the xiphoid process of the sternum, at the level of the solar plexus, identify that "bellows" which is the diaphragm muscle?*

*Can I, by making the sound "a" during exhalation, recognize the vibration being distributed throughout the chest reaching the contact points on the floor?*

*What is alive in me in one movement compared to another?*

*What emotion or thought is associated with a certain gesture and how does it differ, if any, from another?*

The "body that we are" is not rigidly pre-defined by its own "form", it is co-created through the experience we have of the functions it manifests.

In other words, the "idea" I have of the "body I am" changes systematically in relation to the experience I live, moment by moment, through the body itself.

The actions that the body carries out, the movements that it produces and, at the same time, feels and adapts, contribute to creating the internal, internal, cognitive and emotional "image" that we have of the "body that we are".

A significant experience to "sediment" this awareness is the observation of the impact of the expressive motor skills of the face on emotional feeling and therefore, on the retro-active effect that this feeling can have on the "image" I have of the "body that I am".

The experience consists of trying to assume the facial expression that we would assume if we were melancholic and thoughtful and then observing what happens in the rest of the body (how breathing changes, how muscle tone changes, how the hands behave, etc.) and what happens in our emotional feelings.

We remain in observation for the time necessary to recognize the qualities of the experience and then a total state of relaxation of the face is generated, a total detention of the mimic muscles, subsequently opening up to observe what has changed in the body and what has changed in feeling. emotional.

It is common for most people to recognize a notable and significant increase in sensations of physical and emotional discomfort, simply by changing their facial expression "as if" they were feeling melancholy, in the absence of a true etymogenic stimulus that actually produces melancholy.

This correlation allows us to recognize, through direct and immediate experience, how modifying the "function" of a part of the "soma", of the body, of our anatomy, produces a powerful "effect" on our way of hearing and feeling. There.

We know that emotions influence facial expressions but that facial expressions could influence emotions is probably less obvious.

- **Statue-Snail-Jellyfish practice (*noticing, attention regulation, emotional awareness, self-regulation, body listening, trusting, autonomy, self-acceptance, positive relation with others, positive mental health*)**

It is a MB practice performed in pairs.

We find ourselves facing each other. One of the two has his eyes closed and is preparing to receive a series of impulses from his partner.

Those who have their eyes closed are invited, as a first experience, to embody within themselves the qualities of a statue (or even a mountain, an oak, a sequoia): firmness, stability, majesty, but also immobility, stasis, non-deformability.

This is not "pretending to be a statue", but rather trying to find the qualities of the statue in one's own bodily modalities.

After a few brief moments to enter this state, we begin with the actual practice: the partner, with his eyes open, induces pressures and pushes on a part of his partner's body with the aim of gently trying to move it.

Whoever receives the impulse, having his eyes closed, will not be able to anticipate his own reaction as he will not have the opportunity to observe his partner's gestures and will only be able to rely on his own tactile perception. The reaction to the pressure, to the push, must originate from the part of the body touched and not from the entire body as a whole. For example, if the push comes on the front part of the right shoulder, the response must originate from the movement of the shoulder alone and the adjacent districts and not from a global action of forward unbalancing of the whole body.

This first phase of the experience aims to raise participants' awareness of the possibility of "isolating" the kinetic responses of the body to its specific parts, acquiring awareness of the possibility of differentiating and finely modulating neuro-muscular tensions.

An interesting point of this phase is represented by the fact that this reaction to the push, which requires an increase in neuro-muscular tension, is then accompanied by a phase of sudden relaxation following the cessation of the pressure. This offers the participant the opportunity to recognize that flows of tissue tension are constantly present within the body of which we are often unaware.

After this first phase, in which the participants' attention is oriented towards the exquisitely "motor" details of the practice, we enter a phase of observation and listening to the experience with a more emotional value: "*what is alive in the body when the body embodies the qualities of a statue, a mountain, a sequoia? How is your breathing? How is the density of the tissues? How is the state of the viscera? What is the prevailing emotion? What possibilities for dynamism do I have? What advantages/disadvantages does this condition offer me?*"

Evidently, everyone will be able to experience an infinite anthology of answers to these questions. For some, "being a statue" can be associated with a familiar and pleasant condition of presence, for others with a constricting, unnatural and paralyzing condition; for some it can evoke a related feeling of firmness, vividness, power, for others it can evoke embarrassment, rigidity, paralysis.

Obviously, even in this experience, an attitude towards non-judgmental exploration was suggested to the participants: I am not interested in establishing "sentences", but rather in observing the experience and its manifestations.

After embodying the qualities of the statue, a few minutes were given to the person who had undergone the pressure, the pushes, to remain in a relaxed listening state to their body to let the experience settle. The image of following the traces of the practice just carried out was offered: observing the qualities of the body in this moment, the associated thoughts and the emotions present once again not with the aim of judging but, simply, with the desire to " collect” information about yourself.

We then entered the second incarnation: the snail.

By continuing to keep your eyes closed, this time you must embody the qualities of a snail, capable of quickly and vividly retracting when someone touches its "antennae" or "horns". To the touch of its partner, who this time will offer a light contact and no longer a push, the "snail" will have to respond by moving away the part of the body on which the touch arrives. Also in this case, the first phase of the experience has the objective of making participants aware of the possibility of "isolating" the kinetic responses of the body, acquiring awareness of the possibility of responding by retracting, moving away and no longer, as in the case of the statue, resisting, opposing.

In a second time, without interruptions, the participants were invited to observe the emotional qualities of the experience: “*what is alive in the body now that the body embodies the qualities of a snail? How has breathing changed, if it has changed? How is the density of the fabrics now? How is the state of the viscera? What is the prevailing emotion, is it the same as before or different? What possibilities for dynamism do I have? What advantages/disadvantages does this condition offer me?*”

Even in this case, evidently, everyone will be able to experience an infinite range of answers. For some, "being a snail" can be associated with a condition of defeat, discomfort, giving in, for others with a condition of freedom, opportunity, openness.

Again, this phase of incarnation of the snail ended with a time of relaxed observation and sedimentation of the experience following the traces of the practice just carried out: observing the qualities of the body in this moment, the associated thoughts and the emotions present.

The last incarnation: the jellyfish.

The body is now inhabited by the fluid, soft, sinuous, liquid qualities of a jellyfish.

The impulses that are offered by the partner do not produce an equal and opposite response as in the statue, nor a rapid retraction as in the snail.

The impulses “pass through” the body. The body receives the impulse and transforms it into a dynamic movement: a twist, a flexion, a bending, an extension, a combination naturally capable of ensuring that the body knows how to change kinetic shape while maintaining its structural integrity.

It is as if the body were liquid, fluidic, without a rigidly pre-imposed shape and, for this reason, totally capable of acquiring any shape, like water.

*“How is the movement now? How is your breathing now? What are the global sensations of the body? What are the particular sensations of the body? What is the prevailing emotion: is it the same as the two previous experiences or is it different? What advantages/disadvantages does this condition offer me?”*

Again, evidently, the answers could be infinite and infinitely different, however, on the basis of the experience of leading hundreds of groups, the writer can recognize a certain widespread tendency among the participants to associate the jellyfish with a sensation of freedom, pleasantness, relaxation , openness, opportunity, deriving from the ability to recognize in the body an innate tendency towards mutability, a natural predisposition to the transformative multiformity of the body itself which manifests itself in all its adaptable plasticity. This is a condition that the rigid operating schemes that we tend to use often contribute to making us forget.

The last minutes of the practice were characterized by the passage, guided by the teacher, from the "statue" to the "jellyfish", from the "jellyfish" to the "snail", from the "snail" to the "statue" and so on. This sequential experience of a state and its exact opposite (think of the "statue" and the "medusa") is useful for the practitioner to recognize differences through the close experience of the sensorimotor and emotional diversity of the three incarnations.

This is in line with what has been repeatedly stated and associated with Langer's Mindful Learning: "being aware is the simple act of drawing new distinctions. This guides us towards greater sensitivity to context and perspective and, ultimately, greater control over our lives.” Drawing new discriminations while being attentive to the context, the variations, the perspectives, during the learning process, establishes a mindset that is present in detecting possible differences, leading to a conscious awareness (mindful awareness) (Slepian and Ambady, 2012).

The practice ended with a few minutes of listening, relaxed observation and sedimentation of the experience following the traces of the practice: recognizing the sensations of the body, the associated thoughts and the emotions present, allowing the embodied mind to bring out new intuitions within itself and new evolutionary possibilities flourish.

We then moved on to a change of "roles": the one who "suffered" the impulses (i.e. the partner with his eyes closed) now becomes the one who "offers" the impulses and, in the same way, the one who had "offered" the impulses, now he “submits” them with his eyes closed. The sequence is the same as shown in the previous lines.

- **Dialogic practice (*noticing, attention regulation, emotional awareness, self-regulation, body listening, not distracting, autonomy, positive relation with others, personal growth, positive mental health*)**

In the same couples from the previous motor practice, a dialogic sharing practice was then carried out. Compared to dialogic practices which generally take place in groups in mindfulness-based programs, this one has a particular peculiarity: it takes place in pairs with a single interlocutor and not in a group (Khoury *et al.*, 2023).

This is a particularly important moment in which the teacher tried to characterize what Gregory Cramer defines as a "insight dialogue" (Kramer *et al.*, 2023).

What are the reasons for developing a relational awareness meditation?

«In individual practice, over time we develop the ability to let the meditative qualities of the mind-heart “worked” by awareness emerge; qualities that allow a gradual disidentification and the release of the psychic mechanisms and reactivity patterns that habits and experience have sedimented in us. A progressive making ourselves aware and deconstructing the layers of the ego.

However, it is difficult to deny that relationships are the place where emotional reactivity and mental projections are triggered most intensely and easily. In moments in which we find ourselves in relationship with others, our ego activates and manifests its many faces more forcefully.

In addition to being intense, this reactivity is very often conditioned, not intentional.

When language is involved, our attention is almost always "seized" and restricted to the discursive dimension alone, with little or no ability to keep in touch with what is happening in parallel in the rest of the experience, for example at the level of sensations somatic. This increases our vulnerability to automatic, unconscious responses.

How do you bring the meditative quality of awareness to the very moment the relationship happens?

Silent practice is essential, but can often only retrospectively access what we experience in interaction. Furthermore, our familiarity with the individual and silent dimension in which we usually cultivate awareness often leaves us confused and unable to remember to recall it when language comes into play.

Is a form of practice possible that cultivates presence in the very moment of contact with another human being? Which favors the development of meditative qualities and liberating potential in a place so full of conditioned reactivity?

The dialogical practice within the couple sought to cultivate the transfer of the roots of awareness, experienced individually, into a relationship between two.

The setting suggested for the dialogue was deliberately "constructed" with the aim of bringing out some key points useful for subsequent reflections and intuitions.

The members of the couple were seated facing each other, the first of the two to speak had five minutes to share his experience during the experience of the Jellyfish-Snail Statue. The "rule" to be respected was that the "listening" partner could not intervene, but simply listened. After the five minutes of sharing, the "listener" would have three minutes to report to their partner what he had just heard.

Not a mere memorization exercise evidently, rather an excellent opportunity to recognize one's tendency not to listen deeply, to often be inclined to interrupt others, to want to support or correct them, comfort or contradict them.

After reversing the roles, a series of indications were proposed to repeat the experience with operational tools that can facilitate the emergence of a dialogue animated by the flowers of awareness.

The instructions offered, following Gregory Kramer's model, were six:

1. Pause

2. Relax

3. Open

4. Trust in emerging

5. Listen deeply

6. Tell the truth

Divided into three groups, they are easier to implement:

Pause-Relax-Open; Trust in emerging; Listen deeply - Tell the truth.

Each instruction draws on different but complementary meditative qualities. In short:

“Pause” predisposes to awareness; “Relax” opens up tranquility and acceptance; “Open” leads to relational availability and spaciousness; “Trust to emerge” to flexibility and letting go; “Listen deeply” to receptivity and attunement; “Speak the truth” to integrity and care.

«When our consciousness is in direct contact with another consciousness, and both are intentionally placing themselves in the state of conscious presence, it can happen, and in fact it often happens, that a sort of resonance, of mirroring is generated which intensifies the awareness of each, as well as the other qualities of the meditative mind-heart. For example, it is not uncommon for meditators in dyads to experience a level of stability-focus that they report accessing much more rarely in individual practice.

In this sense, relational practice can in certain cases be a sort of accelerator».

- **Free exploration of the floor [Floorwork practice] (*noticing, emotional awareness, self-regulation, body listening, trusting, autonomy*)**

This practice connects to the practice of the Kinesphere.

The participants, this time lying on the ground, were invited to move freely, in a creative and exploratory way, exploiting the dialogue with the floor.

They were invited to explore the various possibilities: sliding, crawling, rolling, pushing, pivoting and every other possible movement mode (Heller-Dani, 2022).

A non-gymnastic attitude was suggested and therefore based on stereotyped movements but rather a truly creative attitude, available to experiment with new or forgotten movements.

It was suggested to try to find fluid movements, avoiding the possibility of breathing becoming blocked in unconscious apneas.

The dialogue between sensorial, motor and emotional dimensions was recalled: *how do I do what I do? What do I feel about doing it? What resistance do I encounter? How can I indulge in curiosity?*

Homework

- **Breathing Awareness Meditation** (20 minutes a day)

An audio guide file was provided with which the participants could carry out this daily practice

- **Floorwork Practice** (15 minutes a day)

**Session 3: 4 hours**

**Presentation**

In this session, after a collective sharing on the personal experience with the practices carried out as homework in the previous days, the participants were offered a detailed description of the most common difficulties, resistances, critical issues in the practice (obstacles) and the related answers and solutions that the Buddhist science of the mind proposes (antidotes).

A review of some of the practices from previous sessions then took place.

**Contents**

- **Sitting Meditation: Breathing Awareness (*noticing, attention regulation, emotional awareness, body listening, not distracting, self-acceptance*)**

As descripted in previous pages.

- **Dialogic practice in group (*noticing, attention regulation, emotional awareness, self-regulation, body listening, not distracting, autonomy, positive relation with others, personal growth, positive mental health*)**

Within a sharing circle, the teacher offered anyone who felt the need to talk about their experience with the practices carried out in the previous weeks, to share their difficulties, their intuitions and any other emerging needs.

The group management method in this phase was the one usually suggested in mindfulness-based programs (for example Mindfulness-Based Stress Reduction).

- **Education in the knowledge of obstacles ad antidotes in the Practices (*autonomy, personal growth*)**

In this part of the session, starting from the previous dialogic phase, we explored the main difficulties and obstacles that can be encountered in the early stages (and not only) of the practice. An exploratory model of obstacles and their antidotes has been illustrated which has roots in the teachings of the Buddhist tradition (Epstein, 1990, Wynne, 2007, Britton *et al.*, 2014, Maitreya, 2000).

*1st Obstacle: Laziness*

It is the most significant obstacle, to which, in fact, four antidotes are associated.

It is a multifaceted condition that should not be associated exclusively with the concept of common laziness. In fact, it is not just a mere lack of will to dedicate oneself to the practice, procrastinating, postponing, finding excuses and justifications. In fact, laziness is also associated with states of surrender and discouragement resulting from a self-judgment which, observing one's difficulties during practice, emerges as an "inevitable" expression from the idea that one must have an ideal performance, a standard to be respected. In fact, in the early stages of the practice, the meaning of the practice itself may not be clear; it can be difficult to understand and embody the "suspension of judgment" and consequently fall into the temptation of wanting to perform. There are frequent feelings of discomfort due to one's inability to maintain stable attention on the body or breathing, for example; it is not clear how the "objective" of the practice is not to aseptically maintain attentional stability, but rather to notice one's own distraction, to become aware of the constant and continuous arising of thoughts, sensations, emotions with which we tend to identify and that prevent us from being totally absorbed in the present moment.

This obstacle, with its many nuances, affects all practitioners at some point in their journey and it is important to know the application strategies of the antidotes.

*Antidotes to Laziness*

- - Trust, Confidence

It is a feeling that progressively and silently matures within oneself through the repetition of practices, through the "study" of one's own mental mechanisms that one experiences firsthand, and not through mere rational and conceptual knowledge. First-person observation of one's own mental dynamics allows one to "collect" data, information and intuitions that help to clarify what previously happened unconsciously. Observing, for example, one's own distraction and being able to return to the object of attention or recognizing one's tendency to "react" rather than "response", are experiences of awareness that lead to the sedimentation of a feeling of trust and confidence. in practice.

By practicing I recognize the benefits of the practice and gain confidence to continue the practice.

- - Aspiration

Aspiration arises on the basis of trust, a powerful feeling capable of directing one's energies towards the desire to cultivate the practice by personalizing its benefits.

The transformations of our interiority do not leave our daily lives, our relationships, our internal dialogue, our behavior indifferent, but rather shape them virtuously and this produces the aspiration to continue the practice.

- - Joyful effort

On the basis of trust and aspiration, a pervasive desire to strive in continuing the practice is generated. An effort that is very different from forcing oneself. It is a support rather than a push, an accompaniment rather than a constraint. And it is a feeling that is supported by joy, that joy of knowing, as we progressively experience it, that we are doing something beneficial for ourselves and for others.

- - Flexibility, ductility

It is a plastic state of soft openness and mental fluidity which is reflected on the physiological, physical and emotional level, which is accompanied by a sort of mental and physical "bliss" and which constitutes a powerful antidote for laziness as it encourages us to continue with vigor practice.

*2nd Obstacle: Forgetfulness*

The second obstacle is represented by forgetting: the object of meditation or the meditation instructions.

A condition, essentially, in which one "gets lost" with respect to the directional intent one would like to pursue.

For example, if the object of meditation is the observation of breathing, forgetfulness is represented by wandering through thoughts, memories, future images, emotions, forgetting that the intent should be to observe breathing.

*Antidote to Forgetfulness*

- - Awareness

Awareness, like trust, aspiration, joyful effort and flexibility, is a state of mind and, as such, presents multiple instances, qualities and constituent elements. One of these, above the others, is memory.

Memory, as an element characterizing awareness, is the quality that neutralizes forgetfulness.

During practice, when I notice a distraction, it is as if I recognize that I have forgotten what I was doing and it causes me to remember the object and the practice instructions. In this sense, awareness is the antidote to forgetfulness.

*3rd Obstacle: Torpor and Agitation*

These are two conditions that tend to disturb the practice and constitute the "detection spectrum" of the subject's energy during meditation. If energy is in excess, we will tend towards agitation, excitement, vice versa, if it is lacking, we will tend towards torpor, drowsiness.

*Antidote to Torpor and Agitation*

- - Meta-Cognition or Introspection

We could consider meta-cognition as that corner of the mind that watches, supervises and checks whether it is necessary to apply an antidote. Once meta-cognition has done its "work" we must abandon it so as not to transcend into a drift of rigid control of practice.

If torpor and agitation appear during practice we can observe them, welcome them as "part" of the experience, let them go or make them an object of practice. If these obstacles are too strong, we can consciously stop the practice.

There are "preventive" strategies that can be useful. If we feel agitated, for example, it could be useful to replace a sitting meditation practice with one of Mindful Movement or to use breathing techniques aimed at lengthening the duration of the expiratory phase (due to its relaxing effects of a parasympathetic nature). If, however, we feel tired, it might be useful to rest before meditating, or take a cold shower, raise the lights in the room, support our posture more vigorously, or use breathing techniques aimed at lengthening the duration of the inspiratory phase (for its activating effects of an orthosympathetic nature).

If torpor and agitation occur recurrently and with similar characteristics from session to session, we can evaluate whether there are more significant upstream factors that may require a specific therapeutic intervention (torpor-depression/agitation-anxiety).

An important aspect is not to forget to be patient with yourself. Any obstacle, including torpor and agitation, is part of our process of inner growth. Encountering obstacles is functional to our flowering process. If there were no obstacles, we would tend towards laziness and complacency.

*4th Obstacle: Not applying the Antidotes*

This is an obstacle whose understanding is rather immediate: despite recognizing what "limits" us in practice and knowing which antidotes we should apply, we do not do so. We are negligent in the application of antidotes.

*Antidote to Do not apply Antidotes*

- - Apply Antidotes

It is clear that, once the obstacle is recognized, in order to remove it, it is necessary to apply the respective antidote. With kindness but equally firmness.

*5th Obstacle: Applying Antidotes too much*

In practice, especially until you have acquired a certain amount of information on the functioning of your mind, you risk being shocked by every little thing that could "disturb" the idea of "perfect" practice that we tend to have. This leads us to want to "control" the practice too rigorously, rigidly, restrictively by applying antidotes reactively.

*Antidote to Over-Applying Antidotes*

- - Equanimity

We could consider equanimity that orienting feeling or attitude that intervenes during practice, allowing us to recognize when an antidote has performed its function and we can let it go by continuing the practice without too much reactivity, rigidity, inflexibility.

- **Free exploration of the floor [Floorwork practice] (*noticing, emotional awareness, self-regulation, body listening, trusting, autonomy*)**

As descripted in previous pages.

- **Kinesphere Practice (*noticing, attention regulation, emotional awareness, body listening, self-regulation, autonomy, environmental mastery, positive mental health*)**

As descripted in previous pages.

Homework

- **Breathing Awareness Meditation** (20 minutes a day)

A new audio guide file was provided with which the participants could carry out this daily practice

- **Floorwork Practice** (10 minutes a day)
- **Kinesphere Practice** (10 minutes a day)

**Session 4: 8 hours**

**Presentation**

The fourth session was focused on the educational in-depth study of fundamental elements for the process of understanding one's own mental mechanisms such as the concept of "autopilot", the difference between the "doing" and "being" modes and the bond between thoughts, emotions and corporeality.

The psycho-educational intervention was accompanied by a series of reflective and dialogic practices that allowed a better and deeper embodiment of the concepts brought to the attention of the group.

**Contents**

- **Sitting Meditation: Breathing Awareness (*noticing, attention regulation, emotional awareness, body listening, not distracting, self-acceptance*)**

As descripted in previous pages.

- **Opening guided Meditation: genesis of a eudaimonic motivation for the day (*attention regulation, emotional awareness, body listening, autonomy, environmental mastery, purpose in lif*e, *positive mental health*)**

After the breathing awareness practice, we continued with this guided practice, in a sitting position, which had as its object of reflection the generation of eudaimonic motivation that could support everyone in the intensive day that was about to begin (Ortner *et al.*, 2018, Kaczmarek, 2017).

First of all, with discernment, one observed one's natural tendency to have hedonic motives (from the ancient Greek ἡδονή *edoné*, "pleasure", or thought that identifies moral good with pleasure, recognizing in it the ultimate goal of the human being).

The most common hedonic motives are pleasure as such, sensory satisfaction, recognition, honors, praise, etc.

More hidden and hidden, however, there are also eudaimonic motives (from the Greek εὐδαιμονία, eudaimonìa der. of εὐδαίμων *eudàimon* «happy», comp. of εὖ «good» and δαίμων «demon; fate»; in philosophical language, happiness understood as fundamental purpose of life). These are motives that emerge upon deeper analysis: the common good, the value of values, compassion, solidarity, the desire to evolve, etc.

From the awareness of these internal "polarities" we have tried to focus on the eudaimonic "pole" of virtuous motivations, trying to enrich the reflection on the good that these motives produce. A choral, collective, shareable and generative good, not merely individualistic, selfish and solipistic.

It was reflected, as always, within a background of openness, lightness and absence of judgement. The objective was not to condemn oneself for one's hedonic motives but rather to recognize that one's hedonic motives can be combined, without opposition, with eudaimonic intentionalities.

- **Guided exercise on the relationship between thoughts, emotions and body sensations (*noticing, emotional awareness, self-regulation, body listening, trusting, autonomy, personal growth, environmental mastery, positive mental health*)**

Participants were asked to close their eyes and imagine this scene:

“*You are walking down the street when on the opposite side of the street you see someone you know but haven't seen for some time; you smile and wave but that person doesn't seem to notice and continues on. How do you feel? What's going through your mind right now? What would you like to do? Are there any bodily sensations coming?”.*

At the end of the exercise, the participants were asked to describe, making a note on a dedicated table, any emotion or physical sensation they felt and any mental image or thought that crossed their mind.

- **Dialogic practice in group (*noticing, attention regulation, emotional awareness, self-regulation, body listening, not distracting, autonomy, positive relation with others, personal growth, positive mental health*)**

Within a circle, after finishing the reflection linked to the previous exercise, we moved on to a moment of sharing. The participants were invited to share what the exercise had evoked in themselves in terms of thoughts, emotions and bodily sensations.

- **Education on the Thoughts-Emotions-Body relationship and the automatic functioning mechanisms of the Mind (*autonomy, personal growth, self-acceptance, positive relation with others, positive mental health*)**

The group sharing was the opportunity to introduce the first element of Psycho-Body Education of the day: the ABC Model of Emotions, an "adaptation" of the formalization technique of cognitive assessment proposed by Albert Ellis (Wirga and DeBernardi, 2002, Muran, 1991, Ellis, 2014).

A system to encourage the subject to understand her own cognitive mechanisms for evaluating events.

ABC is an acronym, where A stands for antecedent, B for belief and C stands for consequence. The antecedent is the starting stimulus, the triggering event, the contingent experiential situation, remembered or imagined (as for example in the previous exercise).

Beliefs are the thoughts, the mental constructions that the subject has used to evaluate (positively or negatively) the antecedent. They are associated with emotions and manifest physiological bodily events (tension, tremor, excitement, tachycardia, numbness, dizziness, etc.).

Finally, consequences can be actions and behaviors.

The previous exercise, experienced in a group dimension, allowed us to appreciate the multiple and diversified emotional, bodily and thought reactions induced by imagining ourselves within that specific situation.

The possibility of starting from the observation that everyone has had different experiences offered the opportunity to focus on a key point: it is not what happens as such that determines our reactions, but rather our interpretations of what happens.

This influences both behavior and the state of the body.

A given triggering situation always and systematically ignites a process in which thoughts and emotions are generated that influence each other.

This thought-emotion cycle manifests itself in a series of sensory and perceptive correlates at a physiological (body) level that support the cycle itself.

From this relational dialogue thoughts-emotions-body our behavior of reaction or response to the triggering situation is produced.

Why is it so interesting to know this interpretative model?

To understand the impact of thoughts on one's behavior in response to a given "trigger" event.

The thoughts we generate in relation to an event that is happening are not "facts", they are mere interpretations, points of view, possible perspectives but, in no way, do they constitute a tangible reality.

Furthermore, thoughts are extremely influenced by emotions.

So it is possible that given the same triggering situation, different emotional substrates produce different thoughts.

Similarly, thoughts of different "colors" can ignite emotions of different "colors" (think of how the thought "I've really gotten older" can ignite a very different emotion from the thought "he definitely didn't see me because he was distracted") .

The response to an event, being influenced by the thoughts-emotions-body triad (Figure 2), can be reactive, stereotyped and reflect old habitual patterns of the mind.

The purpose of this exercise was to understand, in an experiential and not merely conceptual way, that thoughts are not facts.

Often, however, due to our lack of awareness of the functioning processes of the mind, we tend to identify with thoughts, to merge with the thoughts themselves, forgetting that thoughts are thoughts, not reflections of reality.

This is a first key point that invites us to recognize the need to cultivate, within our practices, an attitude towards decentralization, disidentification from thought, and cognitive defusion.

Decentralization is not the act of banishing thoughts, but rather the awareness that we can look at thoughts from a broader perspective, that we can detach ourselves from the contents of thoughts.

Decentralization consists in the ability to modify the relationship with thoughts without touching the content of the thoughts.

The ability to shift the experiential perspective from a total identification with one's own thought contents towards a more open and broader dimension of experience is fundamental to being human (Bernstein *et al.*, 2015, Schooler, 2002, Smallwood and Schooler, 2015).

The part of Education on the Thoughts-Emotions-Body relationship and the automatic functioning mechanisms of the mind continued with the description of the concept of "autopilot" and "living in the head".

The image of the autopilot is evocative.

Sometimes behind the wheel of the car, we drive for miles without really being aware of what we are doing.

Similarly, it may happen that we are not really "present", moment by moment, in many circumstances of our lives: we are often "miles away" without even knowing it.

With autopilot it is more likely that the "buttons" of our automatisms are constantly pressed: events that happen around us and thoughts, emotions and sensations present in our mind can trigger old thinking habits, which are often not helpful Indeed, they can worsen our mood.

When we operate with autopilot engaged, it is easy to unconsciously slip into “doing mode” and ruminative or brooding thought patterns that can cause us to fall into states of sadness or anxiety.

The habitual way of doing also deprives us of our potential to live more fully.

When we are in the doing mode, we are aware of our experience only conceptually, indirectly, and through the thoughts we think about what we are doing.

This means we can easily get lost in ruminations and worries.

Body awareness provides the opportunity to explore a new attitude for knowing things directly, intuitively and experientially.

Knowledge through experience allows you to be aware of unpleasant experiences without getting lost in ruminative thoughts.

A powerful conditioning that distracts us from being "fully present" in every moment is the personal automatic tendency to judge our experience as not entirely correct in some way: "this is not what should happen, this is not good, this is not what I expected or wanted."

Judgments like that can give rise to blameworthy thought patterns, thoughts about what needs to be changed, about how things could or should be different. Often these thoughts quite automatically take us back to old mental routines.

We can thus lose awareness of the moment, and also the freedom to choose whether an action needs to be taken, and which one, or no action at all.

We can regain our freedom if, as a first step, we simply recognize the reality of our situation, without immediately being gripped by the automatic tendency to judge, resolve, or wish things were different from how they are.

We can transform our experience by "intentionally" bringing attention in a particular way.

Let's start practicing getting out of "autopilot" by intentionally and consciously paying attention to the simple act of breathing, eating, listening, observing, the sensations of the body and all the other aspects of our daily experience.

If we become more aware of our thoughts, our emotions and our physical sensations, moment by moment, we give ourselves a greater possibility of freedom and choice; we don't necessarily have to retrace old "mental ruts" that may have caused us problems in the past.

The aim of the practices we are cultivating is to increase awareness, so that we can respond to situations by making a choice rather than reacting automatically.

We do this by practicing becoming more aware of where our attention is placed, and by moving the focus several times and intentionally (Blackledge, 2007, Carmody *et al.*, 2009, Hayes *et al.*, 2012, Segal *et al.*, 2013, Hölzel *et al.*, 2011, Vago and Silbersweig, 2012, Gusnard, 2005, Jordan, 2003, Deikman, 1982, Goleman, 1980, Shapiro *et al.*, 2006, Teasdale *et al.*, 1995).

- **Breathing Space Meditation (*noticing, attention regulation, emotional awareness, body listening, self-regulation, trusting, not worrying, autonomy, self-acceptance, positive mental health*)**

This practice (Miller *et al.*, 2019, Berk *et al.*, 2019), which constitutes the backbone of the Mindfulness-Based Cognitive Therapy (MBCT) program, aims to bring awareness into everyday life. It offers a quick and effective way to transition into being mode in moments of greatest need through open and focused attention.

First of all, participants were invited to pay attention to their posture and correct it so as to embody a sense of "awakening" and the will to voluntarily direct themselves towards what emerges.

The three phases of the Breathing Space practice then followed:

1. *Becoming aware and noticing what your experience is right now*

We become more aware of how things are in the moment by intentionally taking an upright, dignified posture.

If possible, let's close our eyes. Then, bringing awareness to our inner experience and recognizing it, let's ask ourselves: "What is my experience right now? What thoughts are crossing my mind?".

We recognize thoughts as mental events as best we can and try to put them into words.

*“What emotions are there?”.* Let's turn towards unpleasant or uncomfortable perceptions, recognizing them.

“What physical sensations do I perceive in this moment?”. We carry out a quick reconnaissance of the body to capture any sensations of stiffness or tension, recognizing them as such.

2. *Directing attention to the breath and the sensations of the breath*

We bring attention to the physical sensations of breathing. Let's move on to feeling the breath in the abdomen; we perceive sensations in the abdominal wall that expands when the breath comes in and retracts when the breath goes out. We follow the breath along its entire path in and out of the body and use it to anchor us in the present.

3. *Expanding awareness beyond your breath to also notice sensations of the body*

Let us now broaden the field of awareness to understand the body as a whole: the posture, the facial expression, the internal sensations, in particular those of tension and rigidity.

We understand all these sensations within a broader and more spacious awareness.

As best we can, let's try to bring this extended awareness to the next moments of our day.

Homework

- **Breathing Awareness Meditation** (30 minutes a day)

A new audio guide file was provided with which the participants could carry out this daily practice

- **3 minutes Breathing Space Meditation** (whenever you felt overwhelmed by disturbing thoughts or emotions, during the day)
- **Pleasant events diary**

Participants were given a diary in which to record pleasant events that occurred during the day, learning to become aware of experiences no longer as a whole of emotions, thoughts and sensations, but as a constellation of separate elements.

To try to make the participants' learning experience more fruitful, additional readings were provided on the concepts of "Autopilot", "Living in the Head" and the "ABC Model of Emotional Distress".

**Session 5: 4 hours**

**Presentation**

In this session, after the introductory practice of breath awareness, a dialogic practice of group sharing on the experience of the "diary of pleasant events" took place.

The second part of the meeting was dedicated to an educational intervention on stress.

**Contents**

- **Opening Sitting Meditation: Breathing Awareness (*noticing, attention regulation, emotional awareness, body listening, not distracting, self-acceptance*)**

The simplicity of this practice is important because it immediately reveals the difficulty, we all have in putting aside the way we usually use to observe the breath. A modality that makes us tend towards control, expectation, judgment and which prevents us from observing with "simplicity".

When guiding the practice, the teacher reminded the participants that:

- Breathing takes place in the present and brings us back to the here and now.

- The breath is always there, and it is like an anchor or a refuge to return to.

- Breathing indicates your emotional state.

- Breathing can be a substitute for ruminative or ruminative thinking.

- The breath works on its own, it is not our job to operate or control it.

- **Dialogic practice in group (*noticing, attention regulation, emotional awareness, self-regulation, body listening, not distracting, autonomy, positive relation with others, personal growth, positive mental health*)**

Within a circle, participants were invited to share their personal experience with compiling the diary of pleasant events.

A common theme may be difficulty recognizing and managing thoughts, emotions, and physical sensations.

Of course, it is rare for people to make a distinction between these three aspects of mind-body phenomena.

To help each participant make this distinction, feedback from the diary of pleasant events was used which offered the possibility for group members to reflect on what happened when they tried to record these moments, and to report the thoughts exactly, the emotions and physical sensations present.

To support this process, a blackboard was used to write the answers to this exercise, distinguishing (and listing them separately) the different elements that emerged: *"was it a thought, a physical sensation or an emotion?"*.

One thing that often emerges is how apparently irrelevant moments actually contain elements of which we are not aware. Or how apparently negative events can contain elements of pleasantness.

Another aspect of journaling about pleasant experiences is the ability to directly experience the distinction between thoughts, emotions, and physical sensations. If you are aware that experience can be "analyzed" into these elements, it is easier to see thoughts as thoughts, emotions as emotions, physical sensations as physical sensations. Because it is important? Because it is easier to detach from mental states when they are seen as groups of separate elements rather than as elements that join together in an indistinct “mass” (blob). Finally, this exercise highlights that some people find it particularly difficult to become aware of slight physical sensations. The discovery that the body sends signals to the brain, signals that are usually ignored, is actually of enormous importance. Why? First, because such sensations can be used to recognize subtle changes in emotions. Also, because it encourages people to see that there is an alternative to being lost in their mind. Body awareness helps us experience a different “way” of being. Bringing awareness to a bodily sensation changes the nature of emotional experience and offers more ways to respond to what is in the present moment, such as breathing into it.

- **Stress Education (*self-regulation, body listening, trusting, not worrying, autonomy, personal growth, self-acceptance, positive mental health*)**

In this part of the session, in line with the approach traditionally used within MBSR programs, the concept of stress and its implications in health and pathogenesis were explored (Humphrey, 2003, Kohler *et al.*, 2009, Kumar *et al.*, 2013, DeRosier *et al.*, 2013)*.* In particular, an overview was offered on the definitions and types of stress and stressors, coping strategies, reactivity or response mechanisms and the benefits of a mindful approach to stress in daily living.

- **Individual Balance Practice (*noticing, attention regulation, emotional awareness, self-regulation, body listening, not distracting, autonomy*)**

In this sensorimotor practice, each participant was given a pair of small wooden strips to use to create a “T” or “I” shape.

The "T" shape could be created by grabbing one of the two strips and placing it vertically so as to act as a base for the other which, placed horizontally, would create the "T".

The “I” shape could instead be created by placing both strips vertically, one on top of the other.

Once this shape was found, the objective of the practice was to try to maintain the balance of the two strips while moving in space.

Initially the group was given a few minutes to freely explore this experience, spontaneously identifying their own problem-solving methods.

Next, everyone was invited to observe how he was doing what he was doing.

*Where is my gaze directed? How is my breathing? How is the attitude of the fingers that support the base strip? How is the attitude of the opposite hand? What is alive in me?*

*Can I try slowing down my movement? Can I pay more awareness to my steps as I learned to do in Walking Meditation? Can I make the balance of the slats stable without stiffening the rest of the body? Can I observe my performance “anxiety” and let it go? Can I open myself up to the curiosity of "daring"? Can I try to get to sit on the floor? Can I let any instance emerge and open myself totally to the experience?*

This practice, that could improve the static and dynamic balance of young people (Maiano *et al.*, 2019), is a form of focused attention, which differently from sitting meditation practices, such as awareness of breathing for example, offers greater dynamism and can represent an interesting stimulus for the maturation of a transferable moving awareness, as for all the other Mindful Movement practices, to everyday life.

- **Balance practice in pairs (*noticing, attention regulation, emotional awareness, self-regulation, body listening, not distracting, autonomy, positive relation with others*)**

After the individual Balance practice, a "relational" Balance practice was proposed, in pairs.

The members of the couple were sitting on the ground, facing each other about three meters away.

Between them two thin strings were lying on the ground. Each pair then had a pencil.

The objective of the practice was to find a way to, using the two strings as "rails", get the pencil to your partner.

The only rule was that, under no circumstances, the members of the couple could communicate with each other using words.

Every communication had to be limited to body language, observation of the other, the intuition of what I could do to facilitate the other or make the other facilitate me.

This is a very interesting practice which, through the mediation of play, is capable of bringing everyone into contact with what are often mutual "communication" difficulties.

Not being able to talk to each other can exacerbate some internal movements, some emotional motives which, in a silence of words corroborated by attention to the bodily dimensions of the relationship, can be a harbinger of evolutionary and transformative reflections and intuitions.

To increase the difficulty of the experience, the couples were then asked to find a way to balance the pencil on a vertical wooden strip placed halfway between the two partners.

At the end of the practice, everyone was invited, after relaxing within themselves for a few moments, to observe the emotional, intuitive, reflective and sensorial traces of the game just played, allowing the salient elements derived from the serendipity of the experience to settle.

- **Juggling practice (*noticing, attention regulation, emotional awareness, self-regulation, body listening, not distracting, autonomy, positive relation with others*)**

The participants were offered a "simple" juggling practice (moving two balls with one hand with continuity and fluidity) (Malik *et al.*, 2022).

The objective of this experience was above all to come into contact with a "stressful" game in terms of coordination, attention and emotional difficulties.

Participants were invited to observe with care and kindness their tendency to frustration, self-judgment, boredom and any other emerging emotion or sensation or, vice versa, curiosity, the joy of playing, the pleasantness of the challenge and any another emerging instance.

For those who are already familiar with juggling, more complex evolutions have been proposed, with more balls or with more complex and structured movement sequences.

Homework

- **Breathing Awareness Meditation** (30 minutes a day)

A new audio guide file was provided with which the participants could carry out this daily practice.

- **Footblock balance** (15 minutes a day)

Lying on the ground, place an object (a book, a shoe, a block of those used in yoga) on the sole of your foot and keep it in balance while trying to change body position from supine to prone, using as many solutions as possible.

Carry out the practice with both one lower limb and the other.

- **3 minutes Breathing Space Meditation** (whenever you felt overwhelmed by disturbing thoughts or emotions, during the day)
- **Unpleasant events diary**

As with the homework of the previous session, on this occasion the participants were given a diary in which to record the unpleasant events that occurred during the day, with the aim of "training" themselves to learn even better to become aware of the experiences no longer as a set of emotions, thoughts and sensations, but as a constellation of separate elements.

**Session 6: 4 hours**

**Presentation**

In this session, after the introductory practice of breath awareness, a dialogic practice of group sharing on the experience of the "diary of unpleasant events" took place.

The second part of the meeting was dedicated to an educational intervention on salutogenesis, and a meditation on one's own health resources, followed by a walking meditation practice.

**Contents**

- **Opening Sitting Meditation: Breathing Awareness (*noticing, attention regulation, emotional awareness, body listening, not distracting, self-acceptance*)**

As descripted in previous pages.

- **Dialogic practice in group (*noticing, attention regulation, emotional awareness, self-regulation, body listening, not distracting, autonomy, positive relation with others, personal growth, positive mental health*)**

Within a circle, participants were invited to share their personal experience with compiling the diary of unpleasant events.

- **Education in the salutogenic perspective (*not worrying, autonomy, personal growth, self-acceptance, environmental mastery, sense of coherenc*e, *positive mental health*)**

In this part of the session the teacher offered a space for education and information on the salutogenic perspective.

Salutogenesis constitutes the set of factors - material and immaterial, biological and psychological, values and behavior, individual and relational - that create health.

That is, everything that allows people, even in highly critical situations (trauma, chronic illness, disability, adverse events, precarious socio-economic conditions, etc.), to make informed health choices using resources (internal and external), cultivating and increasing one's resilience and pro-active capacity.

The term Salutogenesis is formed from the Latin word *salus, salutis* = health, and from the Greek word *genesis* = origin, beginning, derivation.

Salutogenesis therefore deals with the "causes", or rather the "sources" of health.

It initiates a new approach to health, with the aim of going beyond the pathogenic, mechanistic, reductionist model, still prevailing, which thinks of health as the absence of disease and focuses on the onset, treatment and prevention of diseases.

However, the salutogenic approach does not represent the downside of the pathogenic approach.

While thinking in a pathogenic way means dealing with the onset, treatment or prevention of diseases, salutogenic thinking instead starts from the assumption that all people are more or less healthy and at the same time more or less sick: it is rather a question of knowing how an individual can become healthier or less ill.

Antonovsky (Lindström and Eriksson, 2005, Benz *et al.*, 2014), in fact, suggests interpreting health according to a "continuum between health and disease" model which positions each person at a specific point on this line at a given moment.

This means that, wherever a person is on the continuum, they will always have resources and opportunities to help them shift their personal condition towards the health pole.

The focus of the salutogenic approach is therefore centered on understanding the conditions and mechanisms that favor improvements in health status, regardless of people's health or disease conditions.

Why do some people develop greater health potential?

The answer that Antonovsky proposes is the following: people remain healthy because they develop what he calls Sense of Coherence (SOC), characterized by a pervasive and lasting sense of confidence in the fact that the world is predictable and that there is there is a high probability that things will turn out as one can reasonably expect.

Predictability, optimism and self-confidence are the central elements of this "feeling" which could more precisely be defined as a "thinking style".

According to the Author's definition, the SOC is a global orientation that expresses the point up to which one has a pervasive, lasting although dynamic, feeling of self-confidence in such a way that:

• the stimuli that derive from the internal and external environments of the individual throughout life are structured, predictable and explainable (understandability);

• the resources to respond to the demands that these stimuli pose are available (addressability);

• these requests constitute challenges worthy of investment and commitment (significance).

In other words, SOC would allow people to feel that the world's challenges can be understood, that the resources to face them are available and usable, that there is a meaning and a necessary motivation to "attack" them.

The SOC expresses the general orientation towards the world and towards one's future, the ability to understand the surrounding reality (cognitive dimension), to elaborate one's life horizon (motivational dimension) and to shape any difficulty (behavioral dimension), any stress factor or tragic episode by taming them through multiple and varied resistance strategies such as General Resistance Resources (GRRs).

In addition to the construct Sense of Coherence, Antonovsky developed, in fact, that of GRRs which refer «to the property of a person or a community to implement positive coping with the stress factors innate to human existence».

The concept of GRRs includes biological (healthy constitution), material (money) and psycho-social (knowledge, self-esteem, social support, homeostatic flexibility, cultural capital, intelligence, etc.) factors that make it easier to consolidate strength of resistance useful for dealing with the problems of human existence.

By "internal" salutogenic resources we mean all the physical, psychic, relational, emotional, mental, cognitive, intellectual and spiritual potential of the individual, which can be activated to improve one's health and the development path of one's human potential.

By "external" salutogenic resources we mean all the economic, social, cultural and environmental potential that belong to the individual's life context and which can be activated by the social subjects that populate it to improve health and the development path of human potential of people and communities.

- **Personal Resources Meditation practice: "guided" reflection on one's personal General Resistance Resources (*attention regulation, emotional awareness, body listening, trusting, not worrying, self-acceptance, autonomy, environmental mastery, positive mental health*)**

With the care to which we have become accustomed, we entered into this formal practice in a sitting position which had, as an object of reflection, the vast world of personal resources through which to welcome and transform (where possible) the events of life.

We started by observing how life constantly offers "unforeseen events" and how these require us to access specific "resources" in order to generate an effective and efficient response.

We started by bringing to mind an episode of fever, from how this "unforeseen" event produced, perhaps without us realizing it, a process of events that led us, with the necessary time, to recovery.

We reflected on the resources that were needed to facilitate healing.

We started by reflecting on "external" resources, those to which we tend to be more inclined to pay attention: the support of a family member for example, the intervention of a doctor, the support of a drug, etc.

Subsequently, reflection was invited on "internal" resources, those of which we are often totally unaware.

Those resources that we can synthetically recognize converge in an ancestral "bodily intelligence", capable, for example, of making us recognize those organic sensations different from the natural condition that we usually call "symptoms".

An intelligence that makes us slow down, that invites us to rest.

An intelligence, atavistic and innate, which "reorganizes" and coordinates the metabolic processes of the organism by modulating, like a virtuoso conductor, the chemical reactions, the hormonal responses, the interventions of the immune cells, the conversion of some molecules into others due to the effect of certain enzymes, and the whole wonderful series of events that characterize the healing process.

The same intelligence that, when we recognize the need, leads us to ask for help from "external" resources (the professionalism of a doctor or the support of a family member).

That intelligence which is composed of a multifaceted set of qualities and sub-resources: for example, self-esteem, confidence in one's own psycho-neuro-endocrine-immune resources, self-efficacy, emotional balance, conscious internal dialogue, etc.

Everyone was then invited to recall the memory of their own, further, "difficult" episode, for example a heated argument, a failure at work, failing an exam, or so on.

Having brought that episode to mind (or others, if desired, but being careful not to transcend it into a mere list of points), each person was invited to reflect, with vivid clarity, on all those resources that enable us to face and deal with the stress factors innate to human existence, on their inter-dependence and mutual reciprocity, on the powerful vital energy that can derive from the awareness of being able to access, whatever happens to us, an innate intelligence, a vital nucleus of care that can help us find “healing”.

It was reflected, as always, within a background of openness, lightness and absence of judgement.

The objective, as in all practices, was not to mortify oneself as a result of bringing to mind one or more "difficult" episodes or to enjoy one's own resistance "performances", but rather to recognize how, whatever the difficulty, if we observe ourselves with discernment, an immense potential of resources capable of making us fluidly resilient is alive within us.

- **Walking Meditation (*noticing, emotional awareness, body listening, self-regulation, autonomy, environmental mastery*)**

«Walking meditation is meditation while walking. We walk slowly, in a relaxed way, keeping a light smile on our lips. When we practice this way, we feel deeply at ease and our steps are those of the most confident person on the face of the Earth. All our suffering and anxieties go away, and peace and joy fill our hearts. Everyone can do it. It just takes a little time, a little conscious attention and the desire to be happy».

The practice of walking meditation (Kabat-Zinn, 2017, Edwards *et al.*, 2018, Davis *et al.*, 2022)

is particularly interesting as walking is an action that characterizes large spaces in our daily living.

The group was invited to experience this type of practice in order to recognize how even a single step can constitute a "moment" to return to living awareness of the present moment.

In fact, after having "learned" to stop and become totally present thanks to sitting meditation practices, walking meditation offers the opportunity to enrich Mindful Movement practices through a simple and applicable exercise on all occasions in which one finds oneself walk.

When practicing walking meditation, unlike what usually happens, the steps are not merely functional to arrive at a place, they are themselves the "place" of observation, the "place" of the present.

Our destination is the here and now.

Our destination is here and now.

The practice consists of observing every single step, the sensations of every single step: the contact of the foot, the transmission of the energy of movement within the body, one's balance, one's breathing, and any other emerging sensation.

Each step is supported by a breathing phase.

Inhaling I take a step, exhaling I take another step.

There is synchronization between breathing and steps.

This slow proceeding, marked by the natural flow of breathing, allows us to break the stereotypy, the schematic nature, the unawareness of walking, leaving space for the practitioner to be able to open up to the immense anthology of sensations that each step brings within itself.

This walking, like all other Mindful Movement practices, takes place barefoot, a condition that allows for a more vivid possibility of sensorial listening.

At the beginning, as is normal, this practice can create awkwardness, imbalance and impatience. Each "reaction" that emerges becomes an object of observation and, as with other awareness practices, is let go to return to the observation of the present moment, of the single step, of every single step.

At a later stage, after having cultivated the ability to observe every single step corroborated by a breathing phase, the speed can be increased; for example, in a single inhalation you can "count" four steps, in a single exhalation you can "count" four steps. Counting can constitute the "background" of the experience and facilitate the support of attention that could get lost and distracted.

While walking consciously, breath after breath, a natural process arises in the practitioner of "softening" of resistance, of the will to control, of judgment which facilitates the opening to the "naked" observation of the breath in each step, of each step in the breath. There is nothing in front of me that I have to chase, I simply enjoy every step.

Homework

- **Breathing Awareness Meditation** (30 minutes a day).

Participants were invited to practice independently, without the aid of audio guides.

- **Walking Meditation** (20 minutes a day)

**Session 7+8: 16 hours**

**Presentation**

This session was characterized by offering the group two consecutive days of intensive practice.

Particular emphasis was given to relational motor practices, dialogic practices, the use of voice, bodily expressiveness through mimesis and the experience of forms of meditation of open monitoring and loving kindness.

**Contents**

- **Open Monitoring Meditation: “Bare Attention” (*noticing, attention regulation, emotional awareness, body listening, not distracting, self-acceptance, autonomy, environmental mastery*)**

Bare Attention (Lippelt *et al.*, 2014, Hauswald *et al.*, 2015, Ainsworth *et al.*, 2013) is defined as «the clear and sure awareness of what really happens to us and in us, in subsequent moments of perception». Bare objectless attention does not try to change anything, but observes the mind, emotions and body as they are.

You "enter" this practice by bringing your attention to your breathing, calming your mind to bring it to a deeper state of focus and concentration, ready to engage in meditation.

After a few minutes of centering, altruistic motivation for the meditation session is generated. It is believed that one is not meditating just for one's own individual benefit, but to develop one's mind to become a wiser and kinder person, capable of being of benefit to all beings.

The main part of the practice of Bare Attention begins with focusing attention on the entrance of the nostrils. Paying attention to the very subtle sensations you feel while breathing through your nose.

Everyone will experience certain sensations as the air passes, as the fine hairs move; cooler sensations as you inhale and warmer as you exhale.

You do not follow the air inside the body or out into the surrounding environment. Attention is fixed on the sensations associated with breathing at the entrance to the nostrils.

One must think of oneself as a guardian who simply watches the breath come and go as it enters and leaves the body, carefully observing whatever arises at the point of attention.

At first, if you find it helpful for concentration, you can mentally observe "in" and "out" every time you inhale and exhale.

You use your breath as an anchor for your attention. If the mind continues to wander or is particularly distracted, more emphasis is placed in meditation on the element of concentration by focusing the mind more firmly on the breath.

When you achieve a deeper level of concentration and focus, you loosen your concentration slightly and place more emphasis on the element of awareness in your meditation.

The mind is allowed to be attentive and receptive to whatever it may notice, regardless of what it is.

One does not identify with the mental elements that arise, one simply observes them, like a tourist. You let them arise as you observe them and move on, without getting involved in them. If you are just aware of it.

As you gain more mastery in this type of meditation, each time your mind wanders away from the breath, you begin to take specific note of how and where it has wandered.

Towards thoughts of the past? Towards thoughts of the present or towards fantasies? Towards plans for the future? What type of thoughts tend to attract the attention of our mind? What does this indicate about the afflictions that play a role in our lives?

Having done this for about ten minutes, we take a short break and then resume for another round of about ten minutes and then the meditation session ends with the dedication.

You dedicate all the positive energy that you have created through the practice of mindfulness meditation to becoming an ever better and more at peace with life person, and who can bring more peace and well-being to others at any moment of every day.

The key to the transformative potential inherent in Bare Attention lies in the seemingly simple injunction to separate personal reactions from events themselves.

Most of the time our mind is in a state of reactivity. We take it for granted, we do not question the automatic identifications that we establish with our reactions and we perceive ourselves at the mercy of an often hostile or frustrating external world, or of an uncontrollable and frightening internal world.

With Bare Attention we move from this automatic identification with our fear or frustration to a vantage point from which the fear or frustration is observed with the same dispassionate interest as anything else.

With this change comes immense freedom. Rather than fleeing from painful emotions, or clinging to pleasant ones, the person who practices Bare Attention becomes capable of containing any reaction by making space for it, but not identifying with it completely, thanks to the simultaneous presence of non-judgmental awareness.

- **Practice of Trust (*noticing, emotional awareness, body listening, not distracting, self-acceptance, autonomy, positive relation to others*)**

The group was accompanied in the practice of trusting (Nie, 2018, Rotenberg, 2018, Filkowski *et al.*, 2016): a "game" as a couple in which the objective was to let oneself be led through the space, with one's eyes closed, by one's partner.

The setting: a "driver" and a "passenger", facing each other and the intermediation of a wooden stick to constitute the "bond" between the two companions.

The "driver" begins to move the stick in space, slowly and softly, while the "passenger", listening with his own hand to the end of the stick set in motion by the "driver", responds by following the movement, letting himself be guided, bring, shape in space.

At the beginning the practice takes place on the spot, without moving the feet. The goal is to listen to the experience and observe yourself within the experience: *“Can I let go? Where do I perceive blocks or tensions? Do I apply resistances? How is the quality of my breathing? What is alive in me? Do I tend to judge the driving of the “driver”?”*

Without aiming to control the experience, without falling into the temptation to make the practice a "mechanical" exercise, each was invited to try to let emerge trust in the other's leadership, trust in being able to respond with spontaneous movements devoid of reactivity and control, trust in being able to trust another.

Trust in being able to let go of the breath, trust in being able to let my body explore spaces and angles to which I am not accustomed in my solipsistic movements, trust in being able to grant the other the possibility of flexing me, extending me, making me rotate, bend, etc. trust that the other will be able to guide me ethically.

After this first part of getting in touch with the practice, the possibility of moving in space was offered, of being able to move one's feet to explore the experience.

This is a passage of great intensity: letting the other take me into space, without me seeing where I am going, requires a particular "tact" on the part of the "driver" and a profound act of "trust" on the part of the "passenger".

I might hit a wall, I might trip on another foot, I might get hit by another stick, I might be, in some way, in “danger.”

With the care of both, the patient empathy of the "driver" and the open trust of the "passenger", the natural rigidity and the equally natural angularity of the initial movements can be transformed into a fluid and graceful dance of two bodies which, despite the rhythms differences, times, dynamics, attitudes and feelings, move together, becoming a single body that pulsates in unison.

*“How do I feel? What I feel? How do my feet move? How is the hand that listens to the stick move? How are the facial muscles? How am I breathing? … Can I remove something that is “too much” in me? Can I add something that is "missing"?”*

Without judgment but with discernment, without the desire to control but with the desire not to hinder the natural emergence of the spontaneity of my movement. Of our movement.

At the end of the practice, a few moments of stasis and silence were given to the couples to let the experience settle, to follow the traces of the practice within their own body, of their own mind-body-heart system.

Then, carefully, after thanking each other for the practice, the roles were reversed: the "driver" became the "passenger" and the "passenger" the "driver".

- **Dialogic practice (*noticing, attention regulation, emotional awareness, self-regulation, body listening, not distracting, autonomy, positive relation with others, personal growth, positive mental health*)**

In the same couples involved in the practice of Trusting, a practice of dialogic sharing was then carried out.

Also on this occasion, as during another session of the program, it was proposed to approach the dialogue considering it a "meditation of relational awareness".

The setting suggested for the dialogue was, this time too, "built" with the aim of bringing out some key points useful for subsequent reflections and intuitions.

As already experienced during the fourth meeting, the members of the couple were seated facing each other, the first of the two to speak had five minutes to share his experience during the experience of Trusting. The "rule" to be respected was that the "listening" partner could not intervene, but simply listened. After five minutes of sharing, the "listener" would have three minutes to report to his partner what he had just heard.

Kramer's six-element model was also proposed in this practice: Pause; Relax, Open; Trust in emerging; Listen deeply; Tell the truth.

After reversing the roles, we moved on to a second sharing in which the instruction was to tell the other what we had seen of him within the practice.

In more detail: the "driver" described to the "passenger" what he had been able to observe about him during the experience and, subsequently, the "passenger" described to the "driver" what he had heard, perceived, experienced in the 'be guided.

Also in this case the time was pre-defined: each had five minutes to share with the other. Differently from the previous sharing, the next phase with the three minute "postponement" was not foreseen.

The indication that was offered to the group was to observe their "being" in listening in a non-reactive way, recognizing the innate tendency to judge the words of others, to want to intervene, to want to explain, to want in any way "enter" the other's flow of speech, failing to "stay".

An orientation intention: observe one's own reactivity trying to create space with respect to it, not sitting at the reaction table, simply listening to the other and, perhaps, learning something about myself... through the images of me offered by the other.

After having reversed the roles in the dialogue, each couple was invited to repeat the practice of Trusting.

- **Practice of Trust after the dialogic practice (*noticing, emotional awareness, body listening, not distracting, self-acceptance, autonomy, positive relation to others, positive mental health*)**

Each couple repeated the practice of Trust following the same organizational methods set out previously.

However, everyone was asked to bring within themselves the intuitions and information sedimented in the dialogic practice they had just carried out.

Without wanting to "control" or "correct" but, once again, trying to recognize what we can let go to bring out a spontaneous movement free from parasites.

Wanting to control movement, as seen in previous chapters, is very different from using sensory discrimination to become aware of the different ways in which we produce sensorimotor actions as a learning tool.

Sensory discrimination, the result of observing and observing oneself, is a learning tool that leads to new and different ways of acting and, therefore, to renewed ways of behaving.

- **Dialogic practice after practice of Trust (*noticing, attention regulation, emotional awareness, self-regulation, body listening, not distracting, autonomy, positive relation with others, personal growth, positive mental health*)**

After having repeated the experience of the practice of Trusting again, each couple was invited to repeat, once again, the dialogic practice of sharing, always considering it a "meditation of relational awareness".

Unlike before, this time only the practice time was defined (fifteen minutes) and no further "rules" were imposed. Each couple had the freedom to decide independently how to use the available time.

The practice ended with a brief moment of silent reflection aimed at allowing the reflections and intuitions that emerged during all the practices carried out to settle.

- **Lighthouse Practice in group (*noticing, attention regulation, emotional awareness, body listening, not distracting, autonomy, not worrying, environmental mastery*)**

It is a practice of space-time orientation by being guided by a sound (Murgia and Galmonte, 2015).

The setting is this: the group of participants (which symbolically represents the boats) is positioned in one point of the gym and everyone has their eyes closed and must remain with their eyes closed for the entire duration of the practice.

The teacher (who symbolically represents the lighthouse in the night) positions himself in another point of the gym rather distant from the "boats".

The "lighthouse" teacher will emit a sound (in our case produced through the contact of two wooden sticks) and all the "boats" will begin to move trying to go in the direction of origin of the sound.

Moving with your eyes closed, in a large group, is a very engaging experience both from a sensorimotor and also from an emotional point of view.

Participants were invited to observe their flows of thought, their emotions and the changes in their movement.

- **Lighthouse Practice in pairs (*noticing, attention regulation, emotional awareness, body listening, not distracting, autonomy, not worrying, environmental mastery*)**

Also in this case, it is a practice of space-time orientation by being guided by a sound, but this time it takes place in pairs.

The setting is this: one of the two companions (who symbolically represents the boat) is positioned in a point of the gym with his eyes closed and will try to remain with his eyes closed for the entire duration of the practice.

The other companion (who symbolically represents the lighthouse in the night) positions himself near him and will have two small wooden sticks at his disposal to make a sound through which the "boat" companion will have to try to follow him.

In each pair, within the group, the "lighthouse" emits its own characteristic sound so that it can be recognized by its "boat" companion.

The objective for each partner with his eyes closed is to try to follow his partner who emits his own characteristic sound during his movements in the gym.

The difficulty is that all the pairs move at the same time and therefore there is a lot of background noise.

This implies that each "boat" will have to filter its own "lighthouse" within the noise created.

This is also a very engaging experience both from a sensorimotor and also from an emotional point of view.

- **Vocalizations and listening to vibrational sensations in the body (*noticing, emotional awareness, body listening, self-acceptance, autonomy, positive relation to others*)**

We often forget it but vocalization is a motor act in all respects.

The voice is motion, action, dynamism and cannot fail to be contacted and involved in a Mindful Movement program (Sauder *et al.*, 2010, Angadi *et al.*, 2019).

In this space of the session the participants were invited to observe the bodily sensations resulting from the emission of sounds with different tones, timbres and frequencies.

In particular, participants were invited to observe the natural vibration distributed in the various parts of the body (head, throat, chest, abdomen, and so on) in order to experience the direct link between sound and sensation.

It was suggested to give oneself the opportunity to face shame and embarrassment and make them the object of practice, observation and reflection.

*How do I feel when I make lower pitched sounds? Higher pitched sounds?*

*What emotional tones are associated with one vowel rather than another.*

*How does the vibration of the sound of the other person passing through my body make me feel?*

The practice continued with a game characterized by making different movements associated with different sounds so as to experience how a certain movement associated with a certain sound rather than another produces different sensations and, perhaps, different emotions or thoughts.

- **Floorball Practice (*noticing, emotional awareness, self-regulation, body listening, trusting, autonomy*)**

This practice is connected to the practice of free exploration of the floor seen above.

Participants lie on the ground with their backs resting on a ball and are invited to move on the ball using its sphericity to explore unusual movements.

Rolling on the ball you must try to contact all parts of the body, trying to develop fluid and connected movements, without jerks.

Initially the movements are rather mechanical and angular and then, through the search for fluidity, they become more harmonious and rounded.

- **Matrix Practice (*noticing, attention regulation, emotional awareness, body listening, not distracting, autonomy, positive relation to others, environmental mastery, positive mental health*)**

This is a sensorimotor (Karpati *et al.*, 2016) practice in pairs.

One of the two companions is in an upright position, with his eyes open and the "rule" that he cannot move his feet from where they are at that moment.

The other companion is in front of him and is holding a wooden stick.

The practice develops in this way: the partner with the stick tries to use it to "hit" his partner who, not being able to move his feet from the ground, will have to find a way to dodge the "hits", using bending movements of the torso, torsion, lateral flexion, and any other necessary movement.

This is a very dynamic problem-solving context that requires considerable attention, reactivity, elasticity, fluidity and harmony in movements, and management of emotions.

Obviously, the movements of the stick are careful, not abrupt and absolutely non-violent. The objective of the practice is to bring out a motor flow in both partners that is more similar to a dance than a fight.

The name "matrix" that we have chosen for this practice is linked to the famous scene from the Hollywood film in which the protagonist, faced with an attack of bullets, begins to move fluidly to dodge them.

Fluidity is the horizon towards which the couple tries to strive together.

- **Steals the handkerchief (*noticing, emotional awareness, body listening, self-regulation, autonomy, positive relation to others, environmental mastery*)**

This is also a practice that, at least initially, is carried out in pairs.

The setting is very simple: both companions have a cloth handkerchief of about 40 cm inserted with one end into the elastic of the trousers while the rest of the handkerchief is dangling along the right thigh.

The objective is, when the teacher tells you, to try to "steal" your partner's handkerchief without having your own stolen.

It is a very dynamic practice that involves multiple motor skills and involves multiple emotional dimensions such as competitive spirit, fear of defeat, frustration, joy, and so on.

Every time a partner manages to steal the handkerchief, he returns it to his partner and we continue.

After practicing in pairs, the same game is played in a group in free-for-all mode.

Every time your handkerchief is stolen by a teammate you exit the game while waiting for the end.

The goal is to try to be the only one to keep your handkerchief.

- **Juggling practice (*noticing, attention regulation, emotional awareness, self-regulation, body listening, not distracting, autonomy, positive relation with others*)**

The participants were presented with the juggling practice already practiced in the previous weeks with the aim of evaluating any progress not only in their sensorimotor coordination but, above all, in emotional regulation when faced with a demanding experience from a cognitive-motor point of view.

Also on this occasion the participants were invited to carefully and kindly observe their tendency towards frustration, self-judgment, boredom and any other emerging emotion or sensation or, vice versa, curiosity, the joy of playing, the pleasantness of the challenge and any other emerging instance.

- **Balance practice in pairs (*noticing, attention regulation, emotional awareness, self-regulation, body listening, not distracting, autonomy, positive relation with others*)**

This is also a practice that the participants have already learned about in the 5th session, and which has been proposed again to evaluate the evolution of their motor behavior and their ability to read through their sensations, emotions and thoughts.

- **Practice of Trust using a ball (*noticing, emotional awareness, body listening, not distracting, self-acceptance, autonomy, positive relation to others, positive mental health*)**

The group was accompanied in a different version of the trust practice seen previously, in this same session.

The initial dynamic is the same: it is a couple's "game" in which the objective was to let your partner lead you through the space, with your eyes closed.

The setting involves, once again, a "driver" and a "passenger", one in front of the other and the intermediation, this time, of a ball to constitute the "bond" between the two companions.

The continuation of the practice is similar to that previously described.

- **Orchestra Conductor (*noticing, emotional awareness, body listening, self-regulation, autonomy, positive relation to others, environmental mastery*)**

The Orchestra Conductor practice is a practice of Mindful Movement in pairs.

One of the two members of the couple embodies the role of the "conductor" and will have the task of inducing, through the touch mediated by a thin wooden stick, movements in the body of his partner who will instead embody the role of the "orchestra".

The "conductor", freely and carefully observing the response of his "orchestra" companion, induces a touch on a part of his companion's body, who will move that part with a spontaneous and harmonious response.

The idea is that each touch induced by the "conductor" represents the homologue of the gesture of a real conductor which is followed by the entry into the melody of an instrument with its own sound.

The "orchestra" companion, just like a real orchestra, will make the part of the body touched enter the "kinetic melody" that is being created, as if that part could move being a sound in harmony with the external touch and with one's own internal feeling.

The result of this practice is a sequence of movements fluidly connected to each other, like a harmony of sounds, in which the "orchestra" relies on the guidance of its "conductor".

The "director" with kindness and attention, empathizing with the movements of his partner, resulting from the response to his own touches, has the responsibility of trying to identify the rhythm of the contacts, their sequence and the relative distribution between the various parts of the partner's body. ' “orchestra”. The "objective" of the "director" is to make the movements of his partner fluid, sinuous, harmonious and to do so he relies on empathetic observation and ethical "guidance".

This is a practice, like all relationship practices, which offers the opportunity to observe the quality of one's "being" in experience: *can I generate a spontaneous gesture in response to the touch of the wand? Can I embody the idea that my body can be an orchestra and as such can generate a "motor melody"? Can I identify if there are rigidities, resistances, inhibitions in the body that I can let go of to make room for greater spontaneity? And again, can I let myself be guided or do I tend to resist? What is alive in me right now? Curiosity or discomfort? Joy or embarrassment? Can I welcome thoughts and emotions and, whatever their nature, try to be the best “orchestra” I can?*

*And if my role is that of "conductor": can I be even more "empathetic" with my "orchestra"? What's the next part I could touch to make my partner's movement flow more harmonious? If I changed the frequency of the touches, how would my partner's response change? What is alive in me at this moment when I have the responsibility to be “ethical” in my driving?*

These are some examples of suggestions with which the teacher accompanied the group within this practice. The developments can potentially be infinite.

At the end of the practice the roles in the couple were reversed and we continued with the same previous methods.

- **Guiding the other's body with your touch (*noticing, emotional awareness, body listening, self-regulation, autonomy, positive relation to others, environmental mastery*)**

In this practice one of the two partner remains with his eyes closed trying to increase his willingness to tactilely awareness to his own body (Hertenstein *et al.*, 2006).

The other partner, through the touch of his own hands, guides his partner's body in space.

For example, through the gentle touch of my hands on your right shoulder, I can guide you forward, right, left, and so on. I can do it more smoothly or more jerkily.

I can then move on to guiding the body through a touch on the head, then on the wrist, then on the pelvis, and so on.

For those who are guided, the difficulty is to trust, let go, show trust towards your partner.

This practice also offers precious opportunities to observe *"how I do what I do": how do I breathe when I'm scared and not in control? How does tension change in the muscles of the face? How are my steps? What is alive in my thoughts and emotions?*

- **Loving Kindness and Compassion Meditations (*noticing, emotional awareness, attention regulation, body listening, not distracting, self-acceptance, autonomy, positive relation to others, purpose in life, positive mental health*)**

These meditations are oriented towards the positive and unconditional valorization of emotional states of kindness and compassion.

Both, whether exercised separately or in association, are an integral part of awareness practices regardless of the different Buddhist traditions (Theravada, Japanese, Chinese Zen) (Bodhi, 2005, Kuan, 2007, Sanharakshita, 2004, Sheng-Yen, 2002, Suzuki, 2011).

Loving-kindness meditation aims to develop an affective state of unconditional kindness towards all people.

Salzberg (Salzberg, 1995) considers it a path of profound spiritual transformation.

«The path begins with cultivating an appreciation of our unity with others through generosity, non-harm, the right word and the right action.

Then, based on these qualities, we purify our minds through the concentration practices of meditation. As we do so, we experience wisdom through the recognition of truth and become deeply aware of the suffering caused by separation, and the happiness of knowing our connection with all beings."

Compassion meditation involves techniques aimed at cultivating compassion, that is, a deep and genuine closeness for those who have been affected by suffering, associated with a sincere desire to alleviate this suffering.

Compassion has also been described as a path to greater awareness.

You can learn to pay attention to the moments when you shut down and contract in the face of suffering, anger, fear, or alienation.

In those moments you are asked to ask yourself what difference empathy, forgiveness, patience and tolerance would make. You cultivate your commitment to addressing your responses of aversion, anger, or intolerance.

With awareness and investigation, you find in your heart the generosity and understanding that allow you to open rather than close."

Loving kindness, also known as *metta* (in pali), comes from Buddhism and refers to a mental state of selfless, unconditional kindness towards all beings.

Similarly, compassion (*karuna*) can be defined as an emotion that arouses “the sincere desire for sentient beings to be free from suffering and the causes of suffering.”

Compassion can be compared to the feeling that a loving mother feels to alleviate the suffering of her child in difficulty, but it is aimed at all beings.

Compassion is a fundamental principle of Buddhist philosophy (it is, of course, emphasized by all major world religions, but Buddhist perspectives on compassion have been most widely considered in psychological literature) and the Dalai Lama defines it as: "a openness to the suffering of others with the commitment to alleviate it".

According to the Oxford English Dictionary, the word "compassion" comes from the Latin *compăti*, meaning "to endure, to suffer together."

In the scientific literature, there appears to be broad consensus that compassion involves feeling for a person who is suffering and being motivated to act to help them.

For example, in his seminal work on human emotions Lazarus defines compassion as: "being moved by the suffering of another and wanting to help him."

Similarly, in a major systematic review of compassion and its evolutionary origins, Goetz et al. they define it as: "the feeling that arises when witnessing the suffering of another and that motivates a subsequent desire for help".

Feldman and Kuyken (Feldman and Kuyken, 2011) describe compassion as: “an orientation of mind that recognizes pain and the universality of pain in human experience and the ability to meet that pain with kindness, empathy, equanimity, and patience.”

The effort by Clara Strauss et al. (Strauss *et al.*, 2016) is interesting and clarifying. to bring together the various definitions and considerations on compassion in a synthetic perspective that ends up qualifying compassion as a «cognitive, affective and behavioral process made up of the following five elements which refer both to compassion towards others and to self-compassion:

1. recognize suffering;

2. understand the universality of suffering in the human experience;

3. feel empathy for the person who suffers and connect with the anguish (emotional resonance);

4. tolerate the uncomfortable feelings aroused in response to the person who suffers (e.g. anguish, anger, fear) thus remaining open and accepting of the person who suffers;

5. take action/act to alleviate suffering.

Loving-kindness and compassion are closely linked to the Buddhist notion that all living beings are inextricably connected.

In the elaborate form of compassionate meditation, the meditator conducts a series of contemplations.

According to Buddhist tradition (Book 1, *uraga vagga* [the book of the serpent], *cunda kammaraputta sutta* [AN 10.176]), at each stage the meditation exercise consists in thinking about specific desires (aspirations) for the other, including following:

- may the person be free from enmity;

- may the person be free from mental suffering;

- may the person be free from physical suffering;

- may the person take care of himself happily.

You can start by first directing these feelings of kindness and compassion towards yourself or others, whichever is easier.

During loving-kindness meditation, the person typically proceeds through a series of stages that differ in the focus of the exercise.

These include:

- focus on yourself;

- focus on a good friend (i.e. a person who is still alive and who does not evoke sexual desires);

- focus on a neutral person (i.e. a person who generally does not arouse particularly positive or negative feelings but who is commonly encountered during a normal day);

- focus on a "difficult" person (i.e. a person who is typically associated with negative feelings);

- focus on yourself, on the good friend, on the neutral person and on the difficult person (with your attention equally divided between them);

- ultimately, focus on the entire universe.

As can be seen from this sequence, typically, feelings of gentle warmth and compassion are initially directed towards oneself and then extended to an ever-widening circle of others, finally radiating them in all directions (north, south, east, west and so on), although the order can be changed to suit individual preferences.

Each practice, whether loving-kindness or compassion, whether experienced separately or in combination, represents an exercise that employs the imagination or actual experience of the emotional state as the object of mindful attention and awareness (Hutcherson *et al*., 2008, Carson *et al.*, 2005, Fredrickson *et al.*, 2008).

The practices, in fact, should not be seen as simple mechanical repetitions of images or phrases.

Rather, by consciously investigating what happens when we attempt to generate loving-kindness or compassion, we give ourselves the opportunity to gain knowledge of the nature of these same emotions, as well as our own personal relationships with them.

- **Move the other person's body at a distance (*noticing, emotional awareness, body listening, environmental mastery, positive relation to others*)**

It is a practice in pairs that involves a certain amount of imagination associated with motor action (Lafleur *et al.*, 2002, Guillot *et al.,* 2021).

The two companions are facing each other at a distance of about 2 and a half meters.

One of the two looks at a part of the partner's body as a signal that that is the part of the body he wants to move and then associates an action, for example I can look at your shoulder and start moving it, at a distance, as if the index finger my hand could push it back, then move it to the right, then down and so on.

The partner imagines that the index finger is actually resting on the shoulder and moves accordingly.

After a few seconds, at the discretion of the partner who moves the index finger, we will move on to another part of the body and another way of moving it, for example the gaze can move to the forehead and, this time, instead of moving that part to distance using the index finger, he will be able to use both hands as if they were grasping a sphere and his partner lets him move "as if" his hands were actually resting on his head.

This practice that combines movement and imagination is an excellent tool for observing how different kinesthetic sensations can be associated with different intentions (the index finger rather than the hands on the sphere) and, obviously, these can be accompanied by different emotions and thoughts.

- **Mimesis (*noticing, emotional awareness, body listening, self-regulation, self-acceptance, autonomy*)**

In this practice, which finds its roots in the work of Orazio Costa, an important Italian director and pedagogist, the participants were invited to manifest, through free-expressive and creative movement, the intrinsic qualities of some natural elements (Bonsignore, 2023, Scaramuzzo, 2013, Maggiore and Tedesco, 2023).

People were asked to “become,” with their bodies in motion, a “cloud,” a “tree,” a “fire,” a “stream,” and so on.

Not by theatricalizing the gesture but by trying to embody the qualities of that element: lightness, majesty, greatness, transformability, rigor, exuberance, and so on for each element proposed by the teacher.

For young adults, obviously, this process of mimesis is certainly difficult since many resistances manifest themselves: embarrassment, shame, self-judgement, the suspicious gaze of others. However, we believe that. reclaiming these mimic instincts is extremely healthy for our creative abilities and for re-establishing an empathetic relationship with the environment and with others.

- **Dancing each other's dance (*noticing, emotional awareness, body listening, self-regulation, autonomy, positive relation to others*)**

It is a practice of free expression through spontaneous dance (Bernardi *et al.*, 2017).

One partner is with his eyes closed and the other, with his eyes open, is facing him.

Background music starts and the person with his eyes closed begins to dance freely, without having a choreographic, technical or aesthetic attitude, simply giving himself the opportunity to bring out what the body likes and needs to do.

The partner with his eyes open in front of the "dancer", observes the movements of his partner, the geometries, the rhythms, the emotion that he assumes is associated with him since, when the teacher's command arrives, he will have to act as a "mirror" to your partner.

The idea is that, at the teacher's signal, the "dancer" will reopen his eyes and his "mirror" partner (who will now close his eyes) will propose his own interpretation of the partner's dance.

It's not a question of redoing exactly the same movements with the same order and expressiveness as your partner but trying to give him my personal vision of his dance. I'll show you your dance interpreted by me.

I'll show you how my eyes saw you and how my body, despite all the differences compared to yours, can empathize with you.

At the end of the sharing, roles are changed with a new background song.

- **Pass the cup of water as a group (*noticing, attention regulation, emotional awareness, body listening, not distracting, autonomy, positive relation to others, positive mental health*)**

This is one of the latest motor practices and consists of passing a cup full of water to each other, trying not to let even a drop of water fall.

The cup passes through everyone's hands and the objective is to try to observe what happens: what movement strategy do my classmates use? There are those who wait, those who go towards; there are those who seem scared and those who seem confident. What happens if, instead of distracting myself after my turn has passed, with my intention and my thoughts I try to "support" my partner? Can I sense that this could be an opportunity to experiment with an idea of community cooperation?

- **Meditation on Equanimity and reflection on the conclusion of the program (*noticing, attention regulation, emotional awareness, body listening, trusting, self-acceptance, autonomy, purpose in life, personal growth, positive mental health*)**

After a few minutes of Bare Attention, the group was accompanied in a practice of reflection on the path followed in the previous weeks.

Everyone was invited to observe, in an equanimous way, without the desire to judge, but with the lively discernment that animated the entire journey, themselves within the "journey" experienced together.

Equanimity was taken into consideration, observed and analyzed at length in the first part of this practice.

In fact, it was the attitude characterizing the subsequent reflection and, more generally, equanimity, with its founding elements, constituted one of the attitudes towards which the entire process was aimed.

Equanimity can be defined as a balanced mental state or a dispositional tendency towards all experiences or objects, regardless of their origin or their affective valence (pleasant, unpleasant or neutral) (Desbordes *et al.*, 2014, Baer *et al,* 2008).

Being equanimous means "observing without interfering" within an attitude of "openness and acceptance", not judging the internal experience and not reacting to it.

Equanimity is a state of mental balance, a dispositive tendency towards all experiences or objects, regardless of their affective value (pleasant, unpleasant or neutral) or their origin.

Here we use the term mental balance in its common definition as a state of calm, stability and composure.

Also implied in this definition of equanimity is a level of impartiality such that one can experience unpleasant thoughts or emotions without repressing, denying, judging, or feeling aversion to them.

Grabovac *et al.* they describe equanimity as “approaching pleasant, unpleasant, and neutral experiences with equal interest”.

One of the elements characterizing equanimity, which is perhaps the most significant, is the non-judgmental attitude.

A non-judgmental attitude consists of taking the position of an impartial witness to one's experience. This involves suspending judgment and simply “observing whatever comes up,” including observing one's automatic judgments of “like” or “dislike,” without “judging the judgment.”

It also involves recognizing one's thoughts as "just thoughts" without pursuing them further.

Over the years this process has been called psychological self-distancing, decentering, cognitive defusion, reperceiving, de-reification or meta-cognitive insight.

This shift in perspective in relation to one's experience is not just limited to mindfulness practice, but rather is an extension of a fundamental aspect of psychological development and growth across the lifespan, also known as "the observing self" or as meta-awareness (Ayduk and Kross, 2010, Schooler *et al.*, 2011, Gallagher, 2000, Northoff and Bermpohl, 2004, Northoff *et al.*, 2006, Weber, 2017, Hadash *et al.*, 2016).

This process allows the individual to decouple the directly experienced sensory self from the "narrative" self, the reflective process that maintains continuity of identity over time.

In the absence of meta-awareness, the narrative self and other pervasive cognitive “mental simulation” processes tend to exacerbate emotional responding (e.g., in the form of prolonged sympathetic arousal), cognitive processing, or rumination (Ayduk and Kross, 2008, Kross and Ayduk, 2008).

It is important to emphasize that having equanimity does not mean suppressing emotions or giving up the affective coloring of our life experiences.

Rather, equanimity is an emotion regulation strategy that can change both the magnitude and quality of responses.

After having observed the roots of equanimity with generative attention, the final practice of this eighth meeting continued with self-reflection within the path.

Let's think back to why we came here, to our expectations and to the motivations that pushed us to continue and supported us in cultivating the practices.

What were we hoping to find and in what spirit were we searching for it.

What did we get from the journey, if we got anything, and what did we learn.

What sacrifices have we made, what have we "lost" and what, instead, have we "conquered".

What obstacles have we encountered and what have we learned about ourselves by facing and transforming those obstacles.

What do we carry inside and what horizons do we feel we can explore.

Simply, we observe each reflection, opening ourselves, with equanimity, to the emergence of the experience.

- **Dialogic practice in group (*noticing, attention regulation, emotional awareness, self-regulation, body listening, not distracting, autonomy, positive relation with others, personal growth, positive mental health*)**

In a group, in a choral way, each with their own time and methods, they shared their feelings regarding the program carried out together, based on the guided reflection practice carried out previously.

- **Writing a letter to yourself**

The participants, after the dialogic sharing, were invited to a brief "formal" reflection whose objective was to let settle within themselves what they wanted to be sure of remembering, of bringing into their present and future.

At the end of this space of reflection everyone wrote a letter to themselves that included these aspects and all the others that they considered salient (intentions, hopes, commitments, intuitions, etc.) (Bolton, 2011, Zannini *et al.*, 2011).

The letter was then placed in an envelope, sealed and will be read by everyone at some time in the future.

Homework

- **Freely self-managed practices**

The group was invited to maintain the practice, making it even more their own, returning to the recordings or handouts provided during the meetings, if desired, and cultivating their own path through books, courses, retreats at specialized centers.

BIBLIOGRAPHY

Ayduk, O., Kross, E., 2008. Enhancing the pace of recovery: self-distanced analysis of negative experiences reduces blood pressure reactivity. Psychological science, 19(3), 229–231.

Ayduk, O., Kross, E., 2010. From a distance: implications of spontaneous self-distancing for adaptive self-reflection. Journal of personality and social psychology, 98(5), 809–829.

Ainsworth, B., Eddershaw, R., Meron, D., Baldwin, D. S., Garner, M., 2013. The effect of focused attention and open monitoring meditation on attention network function in healthy volunteers. Psychiatry research, 210(3), 1226–1231.

Anālayo, B., 2019. Meditation on the breath: mindfulness and focused attention. Mindfulness, 10(8), 1684-1691.

Anālayo, B., 2020. Buddhist antecedents to the body scan meditation. Mindfulness, 11(1), 194-202.

Angadi, V., Croake, D., Stemple, J., 2019. Effects of Vocal Function Exercises: A Systematic Review. Journal of voice : official journal of the Voice Foundation, 33(1), 124.e13–124.e34.

Arnold, P. J., 1973. Education and the concept of movement. Bulletin of Physical Education, 9(5), 15.

Baer, R. A., Smith, G. T., Lykins, E., Button, D., Krietemeyer, J., Sauer, S., Walsh, E., Duggan, D., Williams, J. M., 2008. Construct validity of the five facet mindfulness questionnaire in meditating and nonmeditating samples. Assessment, 15(3), 329–342.

Benz, C., Bull, T., Mittelmark, M., Vaandrager, L., 2014. Culture in salutogenesis: the scholarship of Aaron Antonovsky. Global health promotion, 21(4), 16-23.

Berk, L., Veldhuis, M., Hoedemakers, C., van Boxtel, M., van Os, J., 2019. Do brief meditation exercises boost state mindfulness and positive affect in daily life? An experience sampling pilot study. Mindfulness and aging, 77.

Berland, E., 2018. A dramaturgy of embodiment: The study and practice of experiential anatomy. In Physical Dramaturgy (pp. 79-89). Routledge.

Bernardi, N. F., Bellemare-Pepin, A., Peretz, I., 2017. Enhancement of Pleasure during Spontaneous Dance. Frontiers in human neuroscience, 11, 572.

Bernstein, A., Hadash, Y., Lichtash, Y., Tanay, G., Shepherd, K., Fresco, D. M., 2015. Decentering and Related Constructs: A Critical Review and Metacognitive Processes Model. Perspectives on psychological science : a journal of the Association for Psychological Science, 10(5), 599–617.

Blackledge, J. T., 2007. Disrupting verbal processes: Cognitive defusion in acceptance and commitment therapy and other mindfulness-based psychotherapies. The Psychological Record, 57, 555-576.

Block, B. A., 1998. Keep Them in Their “Place”: Applying Laban's Notion of Kinesphere and Place in Teaching Scientific Concepts. Journal of Physical Education, Recreation & Dance, 69(3), 43-47.

Bodhi B., 2005. In the Buddha’s words: An anthology of discourses from the Pali Canon. Wisdom Publications. Boston, MA.

Bolton, G., 2011. Write yourself: Creative writing and personal development. Jessica Kingsley Publishers.

Bonsignore, T., 2023. Come perenne metamorfosi. Orazio Costa e la mimesi. Itinera, 26.

Britton, W. B., Lindahl, J. R., Cahn, B. R., Davis, J. H., Goldman, R. E., 2014. Awakening is not a metaphor: the effects of Buddhist meditation practices on basic wakefulness. Annals of the New York Academy of Sciences, 1307(1), 64-81.

Brown, S. D., Cromby, J., Harper, D. J., Johnson, K., Reavey, P., 2011. Researching “experience”: Embodiment, methodology, process. Theory & Psychology, 21(4), 493-515.

Butler, J. L., 2016. Rediscovering Husserl: Perspectives on the epoché and the reductions. The Qualitative Report, 21(11), 2033.

Carmody, J., Baer, R. A., L B Lykins, E., Olendzki, N., 2009. An empirical study of the mechanisms of mindfulness in a mindfulness-based stress reduction program. Journal of clinical psychology, 65(6), 613–626.

Carson, J. W., Keefe, F. J., Lynch, T. R., Carson, K. M., Goli, V., Fras, A. M., Thorp, S. R., 2005. Loving-kindness meditation for chronic low back pain: results from a pilot trial. Journal of holistic nursing : official journal of the American Holistic Nurses' Association, 23(3), 287–304.

Dambrun, M., Berniard, A., Didelot, T., Chaulet, M., Droit-Volet, S., Corman, M., Juneau, C., Martinon, L.M., 2019. Unified Consciousness and the Effect of Body Scan Meditation on Happiness: Alteration of Inner-Body Experience and Feeling of Harmony as Central Processes. Mindfulness **10**, 1530–1544.

Davis, D. W., Carrier, B., Cruz, K., Barrios, B., Landers, M. R., Navalta, J. W., 2022. A systematic review of the effects of meditative and mindful walking on mental and cardiovascular health. International Journal of Exercise Science, 15(2), 1692-1734.

Deikman, A., 1982. The observing self: Mysticism and psychotherapy. Boston, MA: Beacon Press.

DeRosier, M. E., Frank, E., Schwartz, V., Leary, K. A., 2013. The potential role of resilience education for preventing mental health problems for college students. Psychiatric Annals, 43(12), 538-544.

Desbordes, G., Gard, T., Hoge, E. A., Hölzel, B. K., Kerr, C., Lazar, S. W., Olendzki, A.,Vago, D. R., 2014. Moving beyond Mindfulness: Defining Equanimity as an Outcome Measure in Meditation and Contemplative Research. Mindfulness, 2014(January), 356–372.

Dewey J., 1934 Experience and Education. Collier Books, New York.

Edwards, M. K., Rosenbaum, S., Loprinzi, P. D., 2018. Differential Experimental Effects of a Short Bout of Walking, Meditation, or Combination of Walking and Meditation on State Anxiety Among Young Adults. American journal of health promotion : AJHP, 32(4), 949–958.

Ellis, A., 2014. The revised ABCs of rational-emotive: Therapy (RET). In The evolution of psychotherapy: The second conference (pp. 97-117). Routledge.

Epstein, M., 1990. Psychodynamics of meditation: Pitfalls on the spiritual path. Journal of Transpersonal Psychology, 22(1), 17-34.

Feldman, C., Kuyken, W., 2011. Compassion in the landscape of suffering. Contemporary Buddhism, 12(1), 143-155.

Filkowski, M. M., Anderson, I. W., Haas, B. W., 2016. Trying to trust: Brain activity during interpersonal social attitude change. Cognitive, Affective, & Behavioral Neuroscience, 16, 325-338.

Foucault, M., 1988. Technologies of the self. In Technologies of the self: A seminar with Michel Foucault (Vol. 18, p. 170).

Foucault, M., 2016. L'ermeneutica del soggetto. Corso al Collège de France (1981-1982). Feltrinelli Editore.

Fredrickson, B. L., Cohn, M. A., Coffey, K. A., Pek, J., Finkel, S. M., 2008. Open hearts build lives: positive emotions, induced through loving-kindness meditation, build consequential personal resources. Journal of personality and social psychology, 95(5), 1045–1062.

Gallagher I., I, 2000. Philosophical conceptions of the self: implications for cognitive science. Trends in cognitive sciences, 4(1), 14–21.

Gan, R., Zhang, L.,Chen, S., 2022. The effects of body scan meditation: A systematic review and meta-analysis. Applied psychology. Health and well-being, 14(3), 1062–1080.

Glaser, L., 2015. Reflections on somatic learning processes in higher education: Student experiences and teacher interpretations of Experiential Anatomy into Contemporary Dance. Journal of Dance & Somatic Practices, 7(1), 43-61.

Goleman, D., 1980. A map for inner space. In: Walsh, RN.; Vaughan, F., editors. Beyond ego. Los Angeles, CA: J.P. Tarcher.

Guillot, A., Rienzo, F. D., Frank, C., Debarnot, U., MacIntyre, T. E., 2021. From simulation to motor execution: a review of the impact of dynamic motor imagery on performance. International Review of Sport and Exercise Psychology, 1-20.

Gusnard D. A., 2005. Being a self: considerations from functional imaging. Consciousness and cognition, 14(4), 679–697.

Hadash, Y., Segev, N., Tanay, G., Goldstein, P., Bernstein, A., 2016. The decoupling model of equanimity: theory, measurement, and test in a mindfulness intervention. Mindfulness, 7, 1214-1226.

Haruki, Y., 1996. Comparative and psychological study on meditation. Eburon Publ.

Hauswald, A., Übelacker, T., Leske, S., Weisz, N., 2015. What it means to be Zen: marked modulations of local and interareal synchronization during open monitoring meditation. NeuroImage, 108, 265–273.

Hayes, S. C., Strosahl, K. D., Wilson, K. G., 2012. Acceptance and commitment therapy: The process and practice of mindful change. Guilford press.

Heller-Dani, K., 2022. Dance as movement: meditation root and flow: optimal experiences in floor-work (Bachelor's thesis, University of Malta).

Hertenstein, M. J., Keltner, D., App, B., Bulleit, B. A., Jaskolka, A. R., 2006. Touch communicates distinct emotions. Emotion (Washington, D.C.), 6(3), 528–533.

Hölzel, B. K., Lazar, S. W., Gard, T., Schuman-Olivier, Z., Vago, D. R., Ott, U., 2011. How Does Mindfulness Meditation Work? Proposing Mechanisms of Action From a Conceptual and Neural Perspective. Perspectives on psychological science : a journal of the Association for Psychological Science, 6(6), 537–559.

Hutcherson, C. A., Seppala, E. M., Gross, J. J., 2008. Loving-kindness meditation increases social connectedness. Emotion (Washington, D.C.), 8(5), 720–724.

Humphrey, J. H., 2003. Stress education for college students. Nova Publishers.

Jordan J. S., 2003. Emergence of self and other in perception and action: an event-control approach. Consciousness and cognition, 12(4), 633–646.

Kabat-Zinn, J., 2017. Walking meditations. Mindfulness, 8(1), 249-250.

Kaczmarek, L. D., 2017. Eudaimonic motivation. Encyclopedia of personality and individual differences, 10, 978-981.

Karpati, F. J., Giacosa, C., Foster, N. E., Penhune, V. B., Hyde, K. L., 2016. Sensorimotor integration is enhanced in dancers and musicians. Experimental brain research, 234(3), 893–903.

Kelly, M. G., 2013. Foucault, subjectivity, and technologies of the self. A companion to Foucault, 510-525.

Kohler Giancola, J., Grawitch, M. J., Borchert, D., 2009. Dealing with the stress of college: A model for adult students. Adult Education Quarterly, 59(3), 246-263.

Khoury, B., Manova, V., Adel, L., Dumas, G., Lifshitz, M., Vergara, R. C., Sekhon, H., Rej, S., 2023. Tri-process model of interpersonal mindfulness: theoretical framework and study protocol. Frontiers in psychology, 14, 1130959.

Kramer, Z., Pellegrini, V., Kramer, G., Barcaccia, B., 2023. Effects of insight dialogue retreats on mindfulness, self-compassion, and psychological well-being. Mindfulness, 14(3), 746-756.

Kross, E., Ayduk, O., 2008. Facilitating adaptive emotional analysis: distinguishing distanced-analysis of depressive experiences from immersed-analysis and distraction. Personality & social psychology bulletin, 34(7), 924–938.

Kuan, T. F., 2007. Mindfulness in early Buddhism: New approaches through psychology and textual analysis of Pali, Chinese and Sanskrit sources. Routledge.

Kumar, S., Bhukar, J. P., 2013. Stress level and coping strategies of college students. Journal of Physical Education and Sports Management, 4(1), 5-11.

Lafleur, M. F., Jackson, P. L., Malouin, F., Richards, C. L., Evans, A. C., Doyon, J., 2002. Motor learning produces parallel dynamic functional changes during the execution and imagination of sequential foot movements. NeuroImage, 16(1), 142–157.

Larson G. J. 1987. Introduction to the Philosophy of Sāṃkhya. In Bhattacharya RS, Larson GJ (eds), Encyclopedia of Indian Philosophies, vol.IV, Sāṃkhya: A Dualist Traditionin Indian Philosophy. Motilal Banarsidass, Delhi.

Lindström, B., Eriksson, M., 2005. Salutogenesis. Journal of epidemiology and community health, 59(6), 440–442.

Lippelt, D. P., Hommel, B., Colzato, L. S., 2014. Focused attention, open monitoring and loving kindness meditation: effects on attention, conflict monitoring, and creativity - A review. Frontiers in psychology, 5, 1083.

Liu, Z., Wang, H., Yu, T., Jiao, Y., Zhang, Y., Liu, D., Xu, Y., Guan, Q., Lu, M., 2021. A Review on the Mechanism of Tuina Promoting the Recovery of Peripheral Nerve Injury. Evidence-based complementary and alternative medicine : eCAM, 2021, 6652099.

Maggiore, V., Tedesco, S., 2023. Editoriale: Mimesis come conditio humana. Itinera, 26.

Maïano, C., Hue, O., Morin, A. J. S., Lepage, G., Tracey, D., Moullec, G., 2019. Exercise interventions to improve balance for young people with intellectual disabilities: a systematic review and meta-analysis. Developmental medicine and child neurology, 61(4), 406–418.

Maitreya, A., 2000. Buddha Nature: The Mahayana Uttaratantra Shastra. Written down by Arya.

Malik, J., Stemplewski, R., Maciaszek, J., 2022. The Effect of Juggling as Dual-Task Activity on Human Neuroplasticity: A Systematic Review. International journal of environmental research and public health, 19(12), 7102.

Merleau-Ponty, M.,1962. Phenomenology of Perception (transl. Colin Smith) Routledge & Kegan Paul.

Merleau-Ponty, M., 1964. Signs (trans. Richard C. McCleary) Northwestern University Press. Evanston, IL.

Merleau-Ponty, M., Edie J.M., 1964. The primacy of perception: And other essays on phenomenological psychology, the philosophy of art, history, and politics. Northwestern University Press.

Miller, C. J., Borsatto, J., Al-Salom, P., 2019. Testing a quick mindfulness intervention in the university classroom. Journal of Further and Higher Education, 43(6), 839-847.

Mo, X., Qin, Q., Wu, F., Li, H., Tang, Y., Cheng, Q., Wen, Y., 2021. Effects of breathing meditation training on sustained attention level, mindfulness attention awareness level, and mental state of operating room nurses. American Journal of Health Behavior, 45(6), 993-1001.

Muran, J. C., 1991. A reformulation of the ABC model in cognitive psychotherapies: Implications for assessment and treatment. Clinical psychology review, 11(4), 399-418.

Murgia, M., Galmonte, A., 2015. The role of sound in motor perception and execution. The Open Psychology Journal, 8(1).

Nie, K. D. Y. G., 2018. Trust or defence? The enhancing effect of perceived social mindfulness on cooperative behavior during interactive game. Journal of Psychological Science, (2), 390.

Northoff, G., Bermpohl, F., 2004. Cortical midline structures and the self. Trends in cognitive sciences, 8(3), 102–107.

Northoff, G., Heinzel, A., de Greck, M., Bermpohl, F., Dobrowolny, H., Panksepp, J., 2006. Self-referential processing in our brain--a meta-analysis of imaging studies on the self. NeuroImage, 31(1), 440–457.

Ortner, C. N., Corno, D., Fung, T. Y., Rapinda, K., 2018. The roles of hedonic and eudaimonic motives in emotion regulation. Personality and Individual Differences, 120, 209-212.

Overgaard, S., 2008. How to analyze immediate experience: Hintikka, Husserl, and the idea of phenomenology. Metaphilosophy, 39(3), 282-304.

Rotenberg, K., 2018. The psychology of trust. Routledge.

Rovelli, C., 2014. Sette brevi lezioni di fisica. Adelphi, Milano.

Rovelli, C., 2018. Physics needs philosophy. Philosophy needs physics. Foundations of Physics, 48(5), 481-491.

Rovelli, C., 2020. Helgoland. Adelphi, Milano.

Ryff, C. D., Boylan, J. M., Kirsch, J. A., 2021. Eudaimonic and hedonic well-being. Measuring well-being, 92-135.

Salzberg S., 1995. Loving-kindness. Shambhala, Boston, MA.

Sanharakshita, 2004. Living with kindness: The Buddha’s teaching on metta. Windhorse publications, Birmingham, UK. 2004.

Sauder, C., Roy, N., Tanner, K., Houtz, D. R., Smith, M. E., 2010. Vocal function exercises for presbylaryngis: a multidimensional assessment of treatment outcomes. The Annals of otology, rhinology, and laryngology, 119(7), 460–467.

Scaramuzzo, G., 2013. Mimesis: dalla riflessione teoretica alla prassi educativa. Studi sulla formazione: 16, 1, 2013, 227-238.

Schöne, B., Gruber, T., Graetz, S., Bernhof, M., Malinowski, P., 2018. Mindful breath awareness meditation facilitates efficiency gains in brain networks: A steady-state visually evoked potentials study. Scientific reports, 8(1), 13687.

Schooler J. W., 2002. Re-representing consciousness: dissociations between experience and meta-consciousness. Trends in cognitive sciences, 6(8), 339–344.

Schooler, J. W., Smallwood, J., Christoff, K., Handy, T. C., Reichle, E. D., Sayette, M. A., 2011. Meta-awareness, perceptual decoupling and the wandering mind. Trends in cognitive sciences, 15(7), 319–326.

Segal, Z., Williams, M., Teasdale, J., 2013. Mindfulness-based cognitive therapy for depression. Guilford publications.

Shapiro, S. L., Carlson, L. E., Astin, J. A., Freedman, B., 2006. Mechanisms of mindfulness. Journal of clinical psychology, 62(3), 373–386.

Sheng-Yen, M., 2002. Hoofprint of the ox: principles of the chan buddhist path as taught by a modern Chinese master. Oxford University Press.

Skjaerven, L. H., Mattsson, M., Catalan-Matamoros, D., Parker, A., Gard, G., & Gyllensten, A. L., 2019. Consensus on core phenomena and statements describing Basic Body Awareness Therapy within the movement awareness domain in physiotherapy.

Slepian, M. L., Ambady, N., 2012. Fluid movement and creativity. Journal of Experimental Psychology: General, 141(4), 625.

Smallwood, J., Schooler, J. W., 2015. The science of mind wandering: empirically navigating the stream of consciousness. Annual review of psychology, 66, 487–518.

Strauss, C., Taylor, B. L., Gu, J., Kuyken, W., Baer, R., Jones, F., Cavanagh, K., 2016. What is compassion and how can we measure it? A review of definitions and measures. Clinical psychology review, 47, 15-27.

Suzuki, S., 2011. Zen mind, beginner's mind. Shambhala Publications.

Tavoian, D., Craighead, D. H., 2023. Deep breathing exercise at work: Potential applications and impact. Frontiers in physiology, 14, 1040091.

Teasdale, J. D., Segal, Z., Williams, J. M., 1995. How does cognitive therapy prevent depressive relapse and why should attentional control (mindfulness) training help?. Behaviour research and therapy, 33(1), 25–39.

Vago, D. R., Silbersweig, D. A., 2012. Self-awareness, self-regulation, and self-transcendence (S-ART): a framework for understanding the neurobiological mechanisms of mindfulness. Frontiers in human neuroscience, 6, 296.

Weber, J., 2017. Mindfulness is not enough: Why equanimity holds the key to compassion. Mindfulness & Compassion, 2(2), 149-158.

Wirga, M., DeBernardi, M., 2002. The ABCs of cognition, emotion, and action. Archives of Psychiatry and Psychotherapy, 4(1), 5-16.

Wynne, A., 2007. The origin of Buddhist meditation. Routledge.

Zannini, L., Cattaneo, C., Brugnolli, A., Saiani, L., 2011. How do healthcare professionals perceive themselves after a mentoring programme? A qualitative study based on the reflective exercise of 'writing a letter to yourself'. Journal of advanced nursing, 67(8), 1800–1810.

Zhu, Q., Li, J., Fang, M., Gong, L., Sun, W., Zhou, N., 2016. Effect of Chinese massage (Tui Na) on isokinetic muscle strength in patients with knee osteoarthritis  Journal of traditional Chinese medicine, 36(3), 314–320.
